# Transcatheter Versus Mechanical and Bioprosthetic Surgical Aortic Valve Replacement in Retrospective Patient Cohorts with Aortic Stenosis <75 Years

**DOI:** 10.3390/jcm15145574

**Published:** 2026-07-16

**Authors:** Xi Wang, Lijun Zeng, Yuanweixiang Ou, Xueli Zhang, Yong Peng, Xin Wei, Wei Meng, Yuan Feng, Ole De Backer, Mao Chen

**Affiliations:** 1 Department of Cardiology, West China Hospital, Sichuan University, Chengdu 610044, China; christina.wangxi@foxmail.com (X.W.); ouyuan_william@foxmail.com (Y.O.); pengyong@scu.edu.cn (Y.P.); weizhang_1985812@163.com (X.W.); fynotebook@hotmail.com (Y.F.); 2 Laboratory of Cardiac Structure and Function, Institute of Cardiovascular Diseases, West China Hospital, Sichuan University, Chengdu 610041, China; 3 Cardiac Structure and Function Research Key Laboratory of Sichuan Province, West China Hospital, Sichuan University, Chengdu 610041, China; 4 Department of General Internal Medicine, West China Second University Hospital, Sichuan University, Chengdu 610041, China; june_z@163.com; 5 Key Laboratory of Birth Defects and Related Diseases of Women and Children (Sichuan University), Ministry of Education, Chengdu 610041, China; 6 Health Care Big Data Center of Sichuan Province, Chengdu 610044, China; zhangxueli633@163.com; 7 Department of Cardiovascular Surgery, West China Hospital, Sichuan University, Chengdu 610044, China; meng_wei_1111@yahoo.com; 8 The Heart Centre, Rigshospitalet, Copenhagen University Hospital, 2100 Copenhagen, Denmark; ole.debacker@gmail.com

**Keywords:** aortic stenosis, surgical aortic valve replacement, transcatheter aortic valve replacement, bicuspid aortic valve, China

## Abstract

**Background**: Comparisons between transcatheter and surgical aortic valve replacement (TAVR or SAVR) in younger aortic stenosis (AS) patients are scarce. The aim of the study was to evaluate the 5-year outcomes of AS patients <75 years undergoing TAVR or SAVR. **Methods**: This was a single-center study that retrospectively included AS patients <75 years who underwent transfemoral TAVR or SAVR from a Chinese real-world database (2014–2023). The primary outcome was defined as the composite of all-cause death, stroke, and cardiovascular rehospitalization at 5 years post-procedure. Robust risk adjustment was performed using inverse probability weighting (IPTW), multilevel regression models, and competing-risk analysis. Sensitivity analyses included comparison between TAVR and mechanical or bioprosthetic SAVR separately in the overall cohort and patients with bicuspid aortic valve (BAV). **Results**: A total of 1646 patients undergoing TAVR (*n* = 808) or SAVR (*n* = 838) were finally included. At baseline, TAVR patients had an older age [(67.9 ± 5.2) vs. (56.5 ± 9.6) years, *p* < 0.001] and a higher surgical risk score [(3.1 ± 1.5) vs. (2.1 ± 1.0) %, *p* < 0.001] than SAVR patients, which was well balanced after IPTW. The 5-year adjusted risk of the primary outcome was similar (TAVR 45.9% vs. SAVR 43.4%, weighted hazard ratio, 1.00, 95% confidence interval, 0.64–1.54, *p* = 0.986), which stayed comparable between TAVR and mechanical or bioprosthetic SAVR separately. In BAV patients (*n* = 516), the risk of death was 8.3% in the TAVR group and 3.4% in the SAVR group (*p* = 0.349). The risk of bioprosthetic structural valve deterioration at 5 years was comparable between groups in the overall cohort (5.7% vs. 8.4%, *p* = 0.478) and BAV patients (7.8% vs. 13.2%, *p* = 0.345). **Conclusions**: In this retrospective study of patients aged <75 years, the risk-adjusted 5-year major clinical outcomes were statistically similar between TAVR and SAVR. However, given the inherent historical imbalances, these exploratory findings should be interpreted with caution, and dedicated prospective studies are still needed in younger and BAV populations.

## 1. Introduction

After 20 years’ development, transcatheter aortic valve replacement (TAVR) has evolved into the standard-of-care treatment for elderly patients with severe, symptomatic aortic stenosis (AS) across all surgical risk categories [1,2,3,4,5,6]. The 2020 American College of Cardiology/American Heart Association (ACC/AHA) guidelines for the management of valvular heart disease (VHD) used an age threshold of <65 years to recommend surgical aortic valve replacement (SAVR) and an age >80 years to recommend TAVR [7], while in the recently published (2025) European Society of Cardiology/European Association for Cardio-Thoracic Surgery (ESC/EACTS) guidelines, the age threshold for TAVR has changed to 70 years compared to 75 years in the version of 2021 [8,9].

Severe AS in younger individuals—typically clinically defined as patients aged under 65 or 75 years—presents a distinct and challenging clinical entity. The overall prevalence of AS in the population <65 years is relatively low, estimated at less than 1%, with bicuspid aortic valve (BAV) anatomy accounting for 30–50% of younger AS patients [10]. While SAVR remains the first-line treatment for young AS patients, TAVR may be preferable in individuals presenting with specific high-risk comorbidities such as porcelain aorta, prior chest radiation, or extensive thoracic surgeries; those with a strong preference for a minimally invasive approach to achieve faster recovery, or individuals facing a high risk of future repeat open-heart surgery. Backed by confirmed efficacy and safety by randomized controlled trials (RCTs) in different patient cohorts [1,2,3,4,5,6], there was already a clear trend across the world to adopt TAVR in AS patients <75 years, even in patients <65 years who are potential candidates for mechanical surgical valves, which is outside of current guideline recommendations [11,12]. However, long-term clinical prognosis and valve durability remain the paramount concerns when expanding TAVR indications to this younger population with decades of life expectancy, and more robust evidence is awaited. Additionally, current guidelines show a cautious attitude towards the application of TAVR in patients with BAV anatomy [7,9]. Since the prevalence of BAV anatomy is significantly rising in the younger AS population, the investigation of different procedural strategies for this specific patient cohort is needed.

In this study, we aimed to evaluate the 5-year outcomes of younger (<75 years) AS patients undergoing TAVR or SAVR by using real-world retrospective data, involving both mechanical and bioprosthetic SAVR, also in the aspects of different aortic valve anatomy.

## 2. Methods

### 2.1. Data Sources

We used data from a dedicated VHD database—the percutaneous or surgical treatment of heart valves (PASS) database. The PASS database was established through the collaborations between West China Hospital, Sichuan University in China, and Sichuan Health Care Big Data Center, an authorized government institution. The PASS database contained longitudinal, nonidentified patient electronic health care records collected from patients with VHD in an 8.36 million Chinese population. Diagnoses, procedures, and clinical variables were identified through International Classification of Diseases 10th Revision (ICD-10) codes.

This study received institutional review board approval from West China Hospital, Sichuan University (2023-1085), and was registered in the Chinese Clinical Trial Registry (No. ChiCTR2000038526).

### 2.2. Study Design

Patients diagnosed with severe symptomatic AS by transthoracic echocardiography (TTE), aged 18 to 74 years, with low/intermediate operative risk (Society of Thoracic Surgeons, STS score <8%), who underwent transfemoral TAVR or SAVR—AVR—from 1 January 2014 to 30 June 2023 were retrospectively included in this study. Participants were excluded if there was a prior AVR, severe vascular disease that prevents open-heart surgery or transfemoral TAVR, need for additional mitral/tricuspid valve therapy or root replacement, myocardial infarction or clinical stroke within the last 30 days, current endocarditis, intracardiac tumor, thrombus or vegetation, kidney disease requiring dialysis, or baseline left ventricular ejection fraction (LVEF) <25%.

All baseline and follow-up TTE examinations were performed using standard commercial ultrasound systems by certified, experienced sonographers. To validate data quality and minimize diagnostic bias, all stored digital images were independently reviewed and verified by two cardiologists blinded to the clinical outcomes. Any discrepancies in valve morphology or regurgitation grading were resolved through consensus or arbitration by a third senior imaging expert. Inter-observer and intra-observer variabilities were evaluated in a random sample of 10% of the cohort, demonstrating excellent diagnostic reproducibility.

Due to the increasing use of TAVR over time in this study, the number of patients with long-term follow-up (>5 years) was obviously lower in the TAVR than SAVR group. The risk curves in this study were then all truncated to 5 years (60 months) to narrow this gap.

### 2.3. Study Endpoints

Data for every hospitalization, including diagnoses, were systematically extracted from the PASS database. The rehospitalization counted in this study was defined as the cardiovascular rehospitalization according to the Valve Academic Research Consortium-3 (VARC-3) criteria [13], including procedure-related, valve-related, heart failure-related rehospitalization, and other cardiovascular rehospitalization (e.g., acute myocardial infarction, hypertension, arrhythmia not directly related to the index procedure). The new permanent pacemaker implantation (PPMI) involved the implant within the index hospitalization and rehospitalization after the procedure. Mortality data from the PASS database were verified through the National Household Registration System to add out-of-hospital and out-of-province deaths.

Valve performance-related outcomes included structural valve deterioration (SVD), moderate to severe paravalvular leak (PVL), endocarditis, and valve thrombosis. SVD was only assessed in patients with a bioprosthesis, defined as moderate SVD (mean transprosthetic gradient ≥20 mmHg AND increase ≥10 mmHg from the first follow-up OR moderate intraprosthetic regurgitation) and severe SVD (mean transprosthetic gradient ≥30 mmHg AND increase ≥20 mmHg from the first follow-up OR severe intraprosthetic regurgitation).

The time in therapeutic range (TTR) was assessed for patients undergoing mechanical SAVR by the Rosendaal method, calculated as the total time in therapeutic interval divided by total time of observation. The target international normalized ratio (INR) was pre-set at 1.8–2.5 or 2.0–3.0 [14,15].

The primary outcome was defined as the composite outcome of all-cause mortality, stroke, and rehospitalization at 5 years after the procedure. The secondary outcomes included major clinical outcomes (all-cause mortality, clinical stroke, rehospitalizations), specific rehospitalization, new PPMI, and valve performance at 5 years after the procedure.

### 2.4. Statistical Analysis Plan

Continuous variables were described as mean ± standard deviation (SD) and compared using Student’s *t*-test. Categorical variables were described as percentages (%) and compared using the chi-square test. Time-to-event analyses for all outcomes were originally performed with Kaplan–Meier (KM) estimates and compared with a Log-rank test. During the long recruitment period of this study, clinical guidelines evolved, and both transcatheter and surgical techniques/devices were progressively refined. To mitigate the potential historical bias and period effects, robust risk adjustment and extensive sensitivity analyses were applied. The robust risk adjustment was performed using 2 methods: The primary analysis used multivariable Cox regression, which includes all baseline covariates, generating the unweighted adjusted hazard ratio (aHR) with 95% confidence interval (CI). The secondary analysis was performed using inverse probability of treatment weighting (IPTW), then Cox regression modeling for the composite primary outcome and all-cause death, Fine-Gray time-to-event analysis accounting for competing risk of death for stroke, rehospitalizations, new PPMI, and valve performance; thereafter, a weighted HR with 95% CI was generated for all outcomes. Proportional hazards assumptions were assessed using Schoenfeld residuals for each endpoint. Covariates included in IPTW were patient demographics (age, sex, and body mass index, BMI), underlying medical conditions (history of hypertension, diabetes, coronary artery disease, chronic lung disease, chronic kidney disease, peripheral artery disease, cancer, atrial fibrillation, prior stroke, baseline LVEF, STS score and concomitant coronary artery interventions), and the procedural date to account for the change in AVR approach as well as device generation over time. The balance before and after IPTW was assessed via the absolute standardized mean difference (SMD).

For sensitivity analysis, we used multiple imputations (5 datasets) by chained equations to handle missing covariate data (baseline BMI only; percentage missing: 15.7%) and performed multiple subgroup analyses—aortic valve anatomy and mechanical or bioprosthetic SAVR—to explore the variability in main results. For all subgroup analyses, the IPTW and robust risk adjustment were re-estimated.

All statistical analyses were performed using R software (version 4.4.0, Free Software Foundation, Inc., Boston, MA, USA). IPTW was applied using the R package WeightIt in R software version 4.4.0. Statistical significance was defined as a two-tailed *p*-value of <0.05. No Artificial Intelligence was used in the research and manuscript development.

## 3. Results

### 3.1. Baseline Characteristics

A total of 4772 AS patients who underwent AVR from 1 January 2014 to 30 June 2023 from the PASS database were screened, while 1646 patients were finally included in this study (Supplementary Figure S1). At baseline, patients in the TAVR group (*n* = 808) had an older age [(67.9 ± 5.2) vs. (56.5 ± 9.6) years, *p* < 0.001], a higher STS score [(3.1 ± 1.5) vs. (2.1 ± 1.0) %, *p* < 0.001], and a higher prevalence of underlying diseases than the SAVR group (*n* = 838). After IPTW, the baseline characteristics were well balanced between the two groups, with an SMD of <0.001 across all covariates (Table 1). The mean transvalvular gradient (56.8 ± 14.1 vs. 57.7 ± 15.3 mmHg, SMD 0.064) and the maximal aortic valve velocity (4.8 ± 0.5 vs. 4.9 ± 0.7 m/s, SMD 0.051) at baseline was comparable between groups before IPTW, as well as the prevalence of moderate to severe aortic regurgitation (9.9% vs. 11.9%, SMD 0.065) and mitral regurgitation (2.2% vs. 2.5%, SMD 0.018).

Among patients in the SAVR group, 355 patients (42.4%) were replaced with a bioprosthetic aortic valve, while in the TAVR group, a self-expanding transcatheter heart valve (THV) was implanted in 706 patients (87.4%). The incidence of TAVR use in AS patients <75 years increased from 23% in 2014 to 78% in 2022. While the use of surgical prosthesis stayed similar over the study period, most TAVR patients received the newer generation of domestic THVs after 2021 (Supplementary Figure S2).

### 3.2. Five-Year Outcomes After TAVR or SAVR

**Figure 1:**
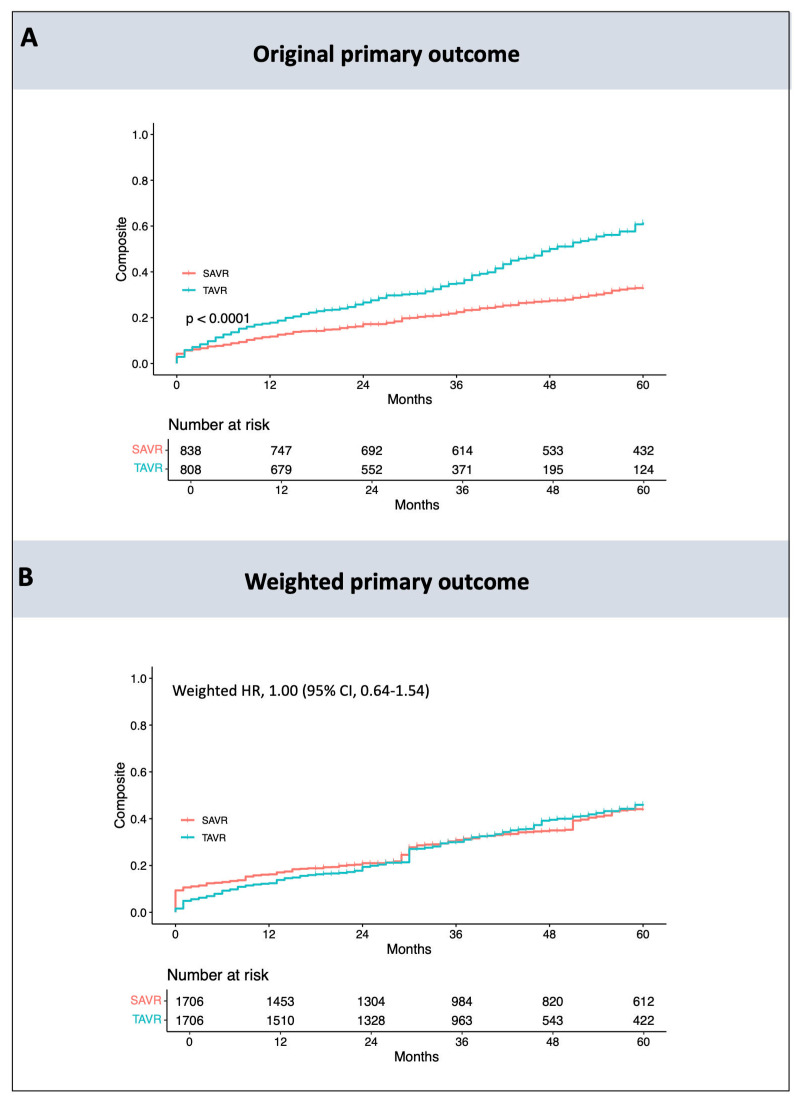
Original and weighted risk of the primary outcome in the overall cohort. Before weighting, the risk of the primary composite outcome (**A**) was significantly higher in the TAVR group than in the SAVR group (Log-rank test, *p* < 0.0001). After weighting, the risk of the primary composite outcome (**B**) was similar between groups (weighted HR, 1.00; 95% CI, 0.64–1.54, *p* = 0.986). SAVR, surgical aortic valve replacement; TAVR, transcatheter aortic valve replacement.

**Figure 2:**
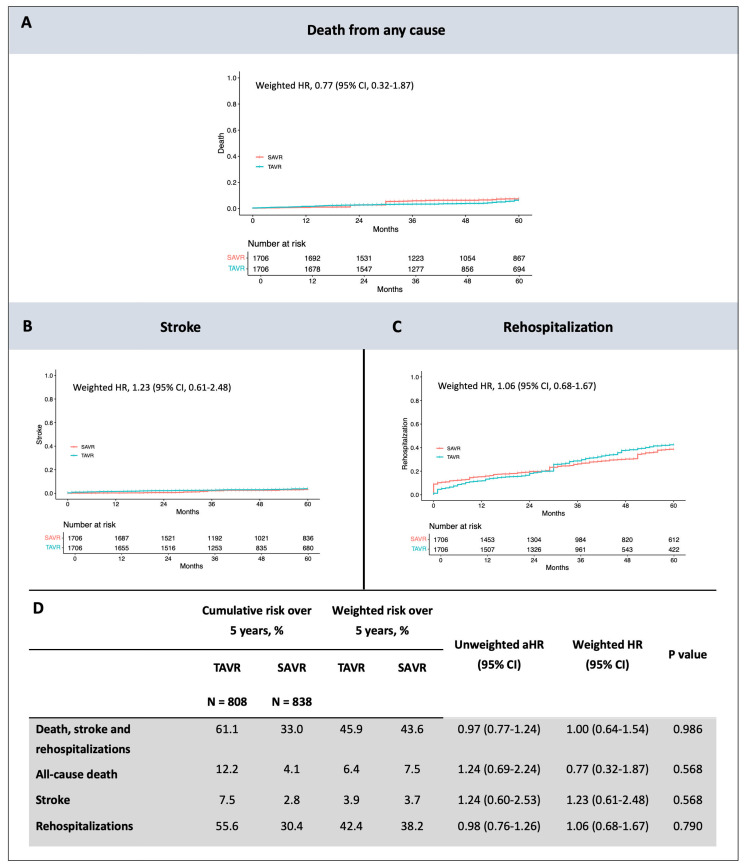
Weighted major clinical outcomes in the overall cohort. The weighted risk of all-cause death (**A**), stroke (**B**), and rehospitalization (**C**) was similar between groups in the overall cohort. A summary of all above outcomes as cumulative risks (Kaplan–Meier estimates), results of robust risk adjustment, and *p*-value for the weighted HR (**D**). SAVR, surgical aortic valve replacement; TAVR, transcatheter aortic valve replacement.

The median follow-up duration for the study population was 56 (IQR: 36–83) months, which was 42 (31–61) months in the TAVR group and 76 (52–97) months in the SAVR group. At 60 months (5 years), the cumulative risk of the composite primary outcome was 61.1% in TAVR group and 33.0% in SAVR group (Log-rank test, *p* < 0.0001) (Figure 1A); however, after multivariable COX regression analysis (unweighted aHR 0.97; 95% CI 0.77–1.24, *p* = 0.825) and after IPTW (weighted risk 45.9% vs. 43.6%, weighted HR 1.00, 95% CI 0.64–1.54, *p* = 0.986), no statistical difference was shown between groups (Figure 1B). The cumulative risk of all-cause death at 5 years was 12.2% and 4.1%, respectively (Log-rank test, *p* < 0.001), with a similar weighted risk after IPTW (6.4% vs. 7.5%, weighted HR 0.77, 95% CI 0.32–1.87, *p* = 0.568) (Figure 2A). In addition, the weighted risk of stroke (weighted HR 1.23; 95% CI 0.61–2.48, *p* = 0.568) and rehospitalization (weighted HR 1.06; 95% CI 0.68–1.67, *p* = 0.790) was comparable in patients undergoing TAVR or SAVR at 5 years after the procedure (Figure 2B–D). Detailed clinical outcomes were shown in Supplementary Figure S3.

The key secondary endpoints were summarized in Table 2. Generally, the weighted 5-year risk of procedure-, valve-, heart failure-related, and other cardiovascular rehospitalization was similar between the TAVR and SAVR groups. The weighted 5-year risk of new PPMI was significantly higher in the TAVR group (22.7% vs. 2.6%; weighted HR, 10.66; 95% CI, 3.34–34.05; *p* < 0.001). Only three TAVR patients and one bioprosthetic SAVR patient underwent aortic valve re-intervention within 5 years after the procedure. The weighted 5-year bleeding risk after the index hospitalization was comparable between groups (4.5% vs. 4.3%; weighted HR, 1.13; 95% CI, 0.51–2.48; *p* = 0.766).

The echocardiographic follow-up was recorded in 1637 patients (99.5%). The mean transvalvular gradient at the latest follow-up was 13.0 ± 6.3 mmHg and 16.2 ± 7.8 mmHg in the TAVR and SAVR group, respectively. At 5 years, moderate-to-severe PVL was rare in both groups but higher in the TAVR group (weighted risk, 6.8% vs. <0.1%; *p* < 0.001; Table 2). Only one TAVR patient presented with valve thrombosis, while nine bioprosthetic SAVR patients, three TAVR patients, and two mechanical SAVR patients presented with infectious endocarditis at 5 years.

### 3.3. Subgroup Analysis

**Figure 3:**
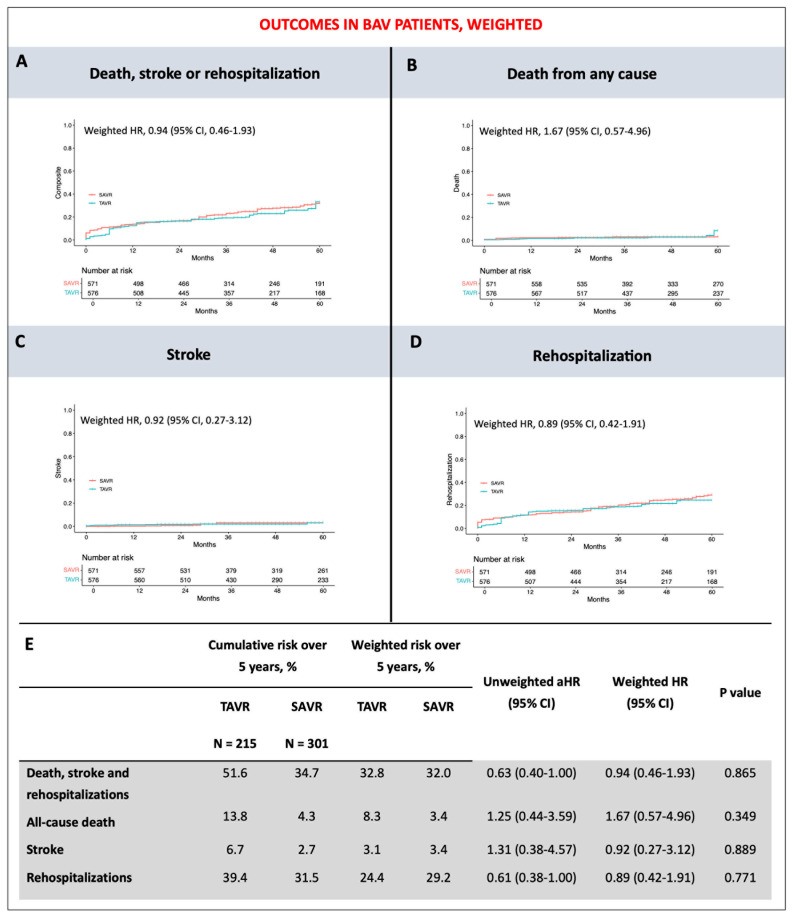
Weighted major clinical outcomes in patients with BAV anatomy. The weighted risk of the primary composite outcome (**A**), all-cause death (**B**), stroke (**C**), and rehospitalization (**D**) was similar between groups in patients with BAV anatomy. A summary of cumulative risks (Kaplan–Meier estimates), results of robust risk adjustment, and *p*-value for the weighted HR in patients with BAV anatomy (**E**). AS, aortic valve; BAV, bicuspid aortic valve; SAVR, surgical aortic valve replacement; TAVR, transcatheter aortic valve replacement.

**Figure 4:**
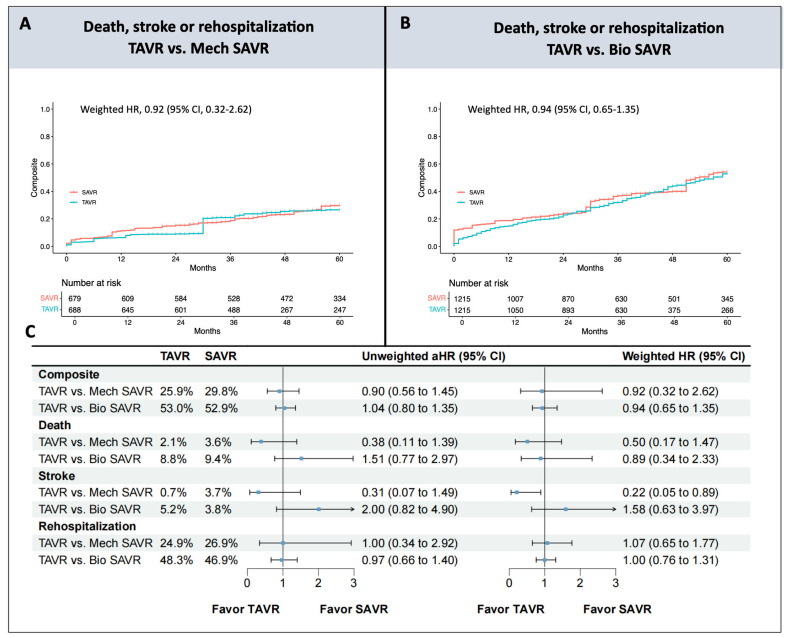
Weighted major clinical outcomes after TAVR vs. mechanical and bioprosthetic SAVR. The weighted risk of the primary composite outcome was similar between the TAVR and SAVR group when the latter group underwent mechanical SAVR (**A**) or bioprosthetic SAVR (**B**). The weighted risk of stroke was lower in the TAVR group than in the mechanical SAVR group (**C**). SAVR, surgical aortic valve replacement; TAVR, transcatheter aortic valve replacement.

Subgroup analyses included the cohort with BAV anatomy (*n* = 516) and TAVR versus mechanical or bioprosthetic SAVR. The comparison between TAVR (*n* = 158) and mechanical SAVR (*n* = 460) was limited to patients <65 years. Generally, the weighted risk of the primary outcome was comparable between groups in the BAV cohort (Figure 3A) and between TAVR and mechanical or bioprosthetic SAVR (Figure 4A,B). Notably, there was no statistical difference on the weighted risk of all-cause death, even though the risk was higher in the TAVR group in BAV patients (8.3% vs. 3.4%, weighted HR 1.67, 95% CI 0.57–4.96, *p* = 0.349) (Figure 3B–E), while the statistical difference was shown comparing TAVR to bioprosthetic SAVR in BAV patients (12.4% vs. 1.3%, weighted HR 5.82, 95% CI 1.53–22.17, *p* = 0.010). The weighted risk of stroke was lower in patients undergoing TAVR than in those undergoing mechanical SAVR (0.7% vs. 3.7%; weighted HR 0.22; 95% CI 0.05–0.89; *p* = 0.034; Figure 4C). TTR for the mechanical SAVR group was 35.7 ± 22.4% (target INR 2.0–3.0) or 50.7 ± 20.3% (target INR 1.8–2.5). The baseline characteristics and detailed outcomes for all subgroups were shown in Supplementary Tables S1–S8 and Supplementary Figures S4–S9.

**Figure 5:**
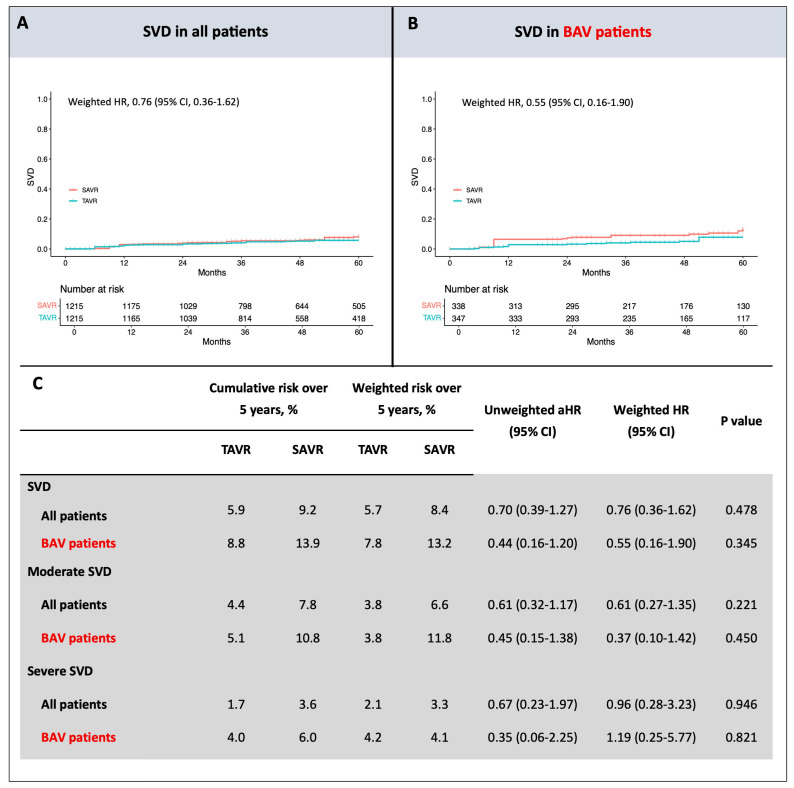
Weighted risk of SVD at 5 years after TAVR vs. bioprosthetic SAVR. The weighted risk of SVD was similar between the TAVR and bioprosthetic SAVR group in the overall cohort (**A**) and patients with BAV anatomy (**B**). A summary of cumulative risks (Kaplan–Meier estimates), results of robust risk adjustment, and p-value for the weighted HR (**C**). BAV, bicuspid aortic valve; SAVR, surgical aortic valve replacement; SVD, structural valve deterioration; TAVR, transcatheter aortic valve replacement.

The weighted risk of SVD was similar after TAVR and bioprosthetic SAVR in the overall cohort and BAV patients (overall cohort: 5.7% vs. 8.4%, weighted HR 0.76, 95% CI 0.36–1.62, *p* = 0.478; BAV anatomy: 7.8% vs. 13.2%, weighted HR 0.55, 95% CI 0.16–1.90, *p* = 0.345). The risk of severe SVD was <5% in both groups at 5 years after the procedure (Figure 5).

## 4. Discussion

In the present study, a 5-year follow-up of AVR in AS patients <75 years was reported. The main findings were as follows: (1) when comparing TAVR to SAVR, or to mechanical/bioprosthetic SAVR separately in AS patients <75 years, the weighted risk of the composite outcome of all-cause death, stroke and cardiovascular rehospitalization was similar between two groups at 5 years, in the overall cohort and in patients with BAV anatomy; (2) analysis of key secondary endpoints showed a higher weighted risk of 5-year all-cause death after TAVR than bioprosthetic SAVR in BAV patients, a lower weighted risk of stroke after TAVR than mechanical SAVR, and generally higher risk of new PPMI and moderate/severe PVL after TAVR than SAVR; and (3) the 5-year incidence of endocarditis, valve thrombosis and re-intervention was very low, while the weighted risk of bioprosthetic SVD was similar between groups in the overall cohort and in patients with BAV anatomy.

A recent meta-analysis of 8 RCTs showed that TAVR was associated with a reduction in the 1-year incidence of all-cause death or any stroke in AS patients at low to moderate risk [16]. The landmark RCTs in the low-risk AS population—NOTION, PARTNER 3 and Evolut Low Risk trials—have also provided solid 5-year comparable clinical safety and efficacy after undergoing TAVR or SAVR [17,18,19]. The latest NOTION-2 trial showed clinical equipoise until 3 years after TAVR vs. SAVR, in a study population with a mean age of 71 years [20,21]. This further promotes the TAVR indication expansion to patients >70 years in the latest ESC/EACTS guidelines [9]. Multiple studies have also shown that the “off-label” use of TAVR in patients <65 years has become common in recent years [22,23,24]. In this study, a patient cohort <75 years was involved, considering the clinical practice in China over the past 10 years; meanwhile, the current guidelines were further explored with a subgroup of patients <65 years.

Real-world 5-year data derived from an authorized, large population-based database were analyzed in this study, showing a higher risk of major clinical endpoints in the TAVR group than the SAVR group. This was mainly due to the obvious baseline difference between groups—much lower age and prevalence of comorbidities in the SAVR group than the TAVR group in the real-world crude data. After applying robust risk assessment and extensive sensitivity analyses, the baseline confounders were well balanced, and the 5-year weighted risk of the composite clinical outcome was comparable between TAVR and SAVR, with the 5-year weighted survival >90% in both groups. In comparison, the NOTION trial 5-year results showed a comparable survival >80% between TAVR and bioprosthetic SAVR in the subgroup of patients <75 years (all-cause death 12.9% vs. 18.5%, *p* = 0.63), while the PARTNER 3 and Evolut Low Risk trial 5-year rate of all-cause death was also similar between groups [17,18,19]. However, given the inherent historical imbalances which cannot be entirely ruled out, the present findings should be interpreted with caution, and dedicated prospective studies are still needed in younger AS populations.

Even though the NOTION-2 trial raised concerns about higher stroke risk after TAVR than SAVR until 3-year follow-up [21], the weighted 5-year risk of stroke was low (around 4%) and similar between groups in this study. A higher risk of new PPMI and moderate/severe PVL after TAVR, compared with SAVR, was seen in the present study, which is consistent with previous findings from RCTs and real-world studies [17,18,19,20,21,25,26]. Importantly, the clinical impact of a higher PPMI rate in this younger patient cohort might be more profound than that in elderly patients, since younger patients with decades of life expectancy face cumulative long-term risks from permanent pacing, including pacing-induced cardiomyopathy, lead-related complications, and the inevitable need for future multiple device changes. While the new PPMI rate is reducing with the continuous improvement of TAVR devices and techniques [27,28], the long-term clinical burden of PPMI remains a pivotal counterweight when considering TAVR over SAVR in young patients.

The 5-year valve performance was also demonstrated, with rare aortic valve re-intervention, and comparable SVD between TAVR and bioprosthetic SAVR (SVD: 5.7% vs. 8.4%; severe SVD: 2.1% vs. 3.3%) in young AS patients. Accordingly, the PARTNER 3 trial 5-year rate of severe SVD was 1.1% and 1.0%, respectively [17], while the latest NOTION-2 trial 3-year results also showed a similar SVD rate (SVD: 4.5% vs. 5.2%; severe SVD: 0.5% vs. 0.0%) between groups [21]. The numerically higher risk of SVD in this study might be related to the absence of routine measurement of bioprosthetic effective orifice area, which also disabled the comparison of prosthesis–patient mismatch between groups. It is worth noting that for low- and intermediate-risk individuals who are younger and have a longer life expectancy, long-term valve durability beyond 10 years plays a crucial role in treatment selection. Long-term prospective data with dedicated echocardiographic follow-up and core-lab assessment are still eagerly awaited to definitively establish the long-term durability profile of TAVR in this population. Furthermore, as bioprosthetic valves will inevitably face failure, the choice between valve-in-valve TAVR and redo SAVR becomes a critical component of lifelong management for younger AS patients, which warrants careful pre-procedural consideration and longer follow-up.

While TAVR is expanding to younger populations, the role of mechanical prostheses cannot be ignored—around 60% of patients in the SAVR group in this study received a mechanical prosthesis. We demonstrated the 5-year weighted risk of clinical outcomes between TAVR and mechanical SAVR in patients <65 years. Interestingly, a higher weighted risk of clinical stroke at 5 years was seen after mechanical SAVR than TAVR. However, the number of events was small in this subgroup analysis, and anticoagulation quality was a major confounder in the mechanical SAVR group given the fact of low TTR. Therefore, this finding should not be interpreted as a definitive advantage of TAVR; instead, the awareness of optimal anticoagulation management should be emphasized in young AS patients undergoing mechanical SAVR.

This study also highlighted that the 5-year weighted major clinical outcomes of younger AS patients with BAV anatomy were statistically similar following TAVR or SAVR. However, a higher weighted 5-year risk of mortality after TAVR could be seen (8.3% vs. 3.4%). This finding was again demonstrated with a statistical difference in TAVR vs. bioprosthetic SAVR in BAV patients. BAV anatomy was identified by transthoracic echocardiography alone in this study; the mortality signal in BAV patients undergoing TAVR may reflect unmeasured anatomical or clinical differences rather than a causal effect. The findings with regard to patients with BAV anatomy were preliminary and exploratory, which should be interpreted with caution. In accordance with current ESC/EACTS and ACC/AHA guidelines [7,9], TAVR may be considered in selected BAV patients (e.g., those with increased surgical risk, suitable anatomy, etc.), while SAVR remains the first-line treatment for young BAV patients. Evidence from dedicated RCTs focusing on younger, lower-risk AS patients with BAV anatomy is warranted, while thorough pre-procedural cardiac CT assessment is critical.

Beyond clinical and anatomical outcomes, the potential expansion of TAVR indications to younger populations carries profound economic and organizational implications for healthcare systems. In younger cohorts, the initial procedure and device cost is compounded by the future financial burden of potential lifetime aortic valve re-interventions and long-term complication management (such as PPMI device replacements). In addition, performing routine TAVR procedures in younger patients demands an expansion of catheterization laboratory capacity and specialized transcatheter heart teams. Cost-effectiveness models must carefully balance these factors before endorsing broad guideline expansions.

## 5. Limitations

The major limitations of this study compared to RCTs and large-scale multi-center registries are its retrospective, single-center design, lack of cardiac CT measurements and independent core-laboratory assessments for valve performance, as well as limited subgroup sample size. During the study timeframe, patient management guidelines underwent several updates, and both transcatheter and surgical technologies were continuously refined. Although we performed rigorous methods to control confounders, the inherent historical bias cannot be entirely eliminated. Additionally, some granular clinical details, including usage of medical therapy, were not fully standardized or available for all individuals, which could be a residual confounding. All post-procedural clinical events were defined according to the rehospitalization records in the database; there was potential under-ascertainment and under-estimation of each clinical endpoint. Pre-procedural cardiac CT was not a mandatory routine in the SAVR group; therefore, detailed aortic valve anatomical features were not available. Finally, this study reported the analysis from a Chinese population-based database; observations from other countries using a national database are warranted to further test the reproducibility.

## 6. Conclusions

Long-term comparative data between TAVR and SAVR in patients <75 years remain scarce. In this retrospective study of patients aged <75 years with lower surgical risk, after robust risk adjustment, 5-year major clinical outcomes were statistically similar between TAVR and SAVR. The 5-year risk of bioprosthetic SVD appeared comparable between groups. However, given the inherent historical imbalances, these exploratory findings should be interpreted with caution, and dedicated prospective studies are still needed in younger and BAV populations.

## Figures and Tables

**Table 1:** Baseline characteristics between the TAVR and SAVR groups, before and after weighting.

	Unweighted			IPTW-Weighted		
	TAVR (*n* = 808)	SAVR (*n* = 838)	SMD	TAVR	SAVR	SMD
Age, years	67.9 ± 5.2	56.5 ± 9.6	1.48	61.9 ± 11.3	61.9 ± 9.1	<0.001
Male	440 (54.5)	420 (50.1)	0.09	55.1	55.1	<0.001
BMI, kg/m^2^	23.3 ± 3.6	23.9 ± 3.2	0.18	23.8 ± 3.3	23.8 ± 3.4	<0.001
STS score, %	3.1 ± 1.5	2.1 ± 1.0	0.75	2.5 ± 1.3	2.5 ± 1.1	<0.001
Hypertension	288 (35.6)	167 (19.9)	0.36	29.4	29.4	<0.001
Diabetes mellitus	139 (17.2)	48 (5.7)	0.37	11.1	11.1	<0.001
COPD	140 (17.3)	8 (1.0)	0.59	8.5	8.5	<0.001
Coronary artery disease	168 (20.8)	67 (8.0)	0.37	15.8	15.8	<0.001
Peri-artery disease	22 (2.7)	1 (0.1)	0.22	3.5	3.5	<0.001
Prior stroke	30 (3.7)	8 (1.0)	0.18	2.1	2.1	<0.001
Atrial fibrillation	94 (11.6)	21 (2.5)	0.36	6.2	6.2	<0.001
Chronic kidney disease	55 (6.8)	5 (0.6)	0.33	3.4	3.4	<0.001
Cancer	20 (2.5)	4 (0.5)	0.17	1.2	1.2	<0.001
LVEF	57.0 ± 14.0	63.4 ± 10.0	0.53	58.6 ± 15.0	58.6 ± 12.4	<0.001
Concomitant CABG/PCI	11 (1.4)	29 (3.5)	0.14	7.7	7.7	<0.001

CABG, coronary artery bypass grafting; COPD, chronic obstructive pulmonary disease; BMI, body mass index; IPTW, inverse probability of treatment weighting; LVEF, left ventricular ejection fraction; PCI, percutaneous coronary intervention; SAVR, surgical aortic valve replacement; SMD, standardized mean difference; STS, Society of Thoracic Surgeons; TAVR, transcatheter aortic valve replacement.

**Table 2:** Key secondary endpoints at 5 years between the TAVR and SAVR groups.

	Cumulative Risk Over 5 Years, %		Weighted Risk Over 5 Years, %		Weighted HR (95% CI)	*p*-Value
	TAVR	SAVR	TAVR	SAVR		
	*n* = 808	*n* = 838				
Heart failure rehospitalizations	23.8	7.4	13.3	10.2	1.17 (0.58–2.36)	0.662
Procedure-related rehospitalizations	14.1	6.6	12.6	12.1	1.18 (0.48–2.88)	0.714
Valve-related rehospitalizations	8.2	5.3	5.0	6.2	0.81 (0.39–1.67)	0.567
Other CV rehospitalizations	8.2	10.9	8.1	9.7	0.81 (0.45–1.44)	0.467
Bleeding	7.5	4.2	4.5	4.3	1.13 (0.51–2.48)	0.766
New permanent pacemaker implant	18.2	2.0	22.7	2.6	10.66 (3.34–34.05)	<0.001
Moderate/severe paravalvular leak	6.1	0.14	6.8	<0.1	91.38 (11.21–744.73)	<0.001

CI, confidence interval; CV, cardiovascular; HR, hazard ratio; SAVR, surgical aortic valve replacement; TAVR, transcatheter aortic valve replacement.

## Data Availability

The data underlying this article will be shared on reasonable request to the corresponding author.

## Supplementary Materials

The following supporting information can be downloaded at: https://www.mdpi.com/article/10.3390/jcm15145574/s1.

## References

[1] Leon M.B., Smith C.R., Mack M., Miller D.C., Moses J.W., Svensson L.G., Tuzcu E.M., Webb J.G., Fontana G.P., Makkar R.R. (2010). Transcatheter aortic-valve implantation for aortic stenosis in patients who cannot undergo surgery. N. Engl. J. Med..

[2] Smith C.R., Leon M.B., Mack M.J., Miller D.C., Moses J.W., Svensson L.G., Tuzcu E.M., Webb J.G., Fontana G.P., Makkar R.R. (2011). Transcatheter versus surgical aortic-valve replacement in high-risk patients. N. Engl. J. Med..

[3] Leon M.B., Smith C.R., Mack M.J., Makkar R.R., Svensson L.G., Kodali S.K., Thourani V.H., Tuzcu E.M., Miller D.C., Herrmann H.C. (2016). Transcatheter or Surgical Aortic-Valve Replacement in Intermediate-Risk Patients. N. Engl. J. Med..

[4] Reardon M.J., Van Mieghem N.M., Popma J.J., Kleiman N.S., Søndergaard L., Mumtaz M., Adams D.H., Deeb G.M., Maini B., Gada H. (2017). Surgical or Transcatheter Aortic-Valve Replacement in Intermediate-Risk Patients. N. Engl. J. Med..

[5] Mack M.J., Leon M.B., Thourani V.H., Makkar R., Kodali S.K., Russo M., Kapadia S.R., Malaisrie S.C., Cohen D.J., Pibarot P. (2019). Transcatheter Aortic-Valve Replacement with a Balloon-Expandable Valve in Low-Risk Patients. N. Engl. J. Med..

[6] Popma J.J., Deeb G.M., Yakubov S.J., Mumtaz M., Gada H., O’Hair D., Bajwa T., Heiser J.C., Merhi W., Kleiman N.S. (2019). Transcatheter Aortic-Valve Replacement with a Self-Expanding Valve in Low-Risk Patients. N. Engl. J. Med..

[7] Otto C.M., Nishimura R.A., Bonow R.O., Carabello B.A., Erwin J.P., Gentile F., Jneid H., Krieger E.V., Mack M., Writing Committee Members (2021). 2020 ACC/AHA Guideline for the Management of Patients with Valvular Heart Disease: A Report of the American College of Cardiology/American Heart Association Joint Committee on Clinical Practice Guidelines. J. Am. Coll. Cardiol..

[8] Vahanian A., Beyersdorf F., Praz F., Milojevic M., Baldus S., Bauersachs J., Capodanno D., Conradi L., De Bonis M., De Paulis R. (2022). 2021 ESC/EACTS Guidelines for the management of valvular heart disease. Eur. Heart J..

[9] Praz F., Borger M.A., Lanz J., Marin-Cuartas M., Abreu A., Adamo M., Ajmone Marsan N., Barili F., Bonaros N., Cosyns B. (2025). 2025 ESC/EACTS Guidelines for the management of valvular heart disease. Eur. Heart J..

[10] Belluschi I., Buzzatti N., Castiglioni A., De Bonis M., Montorfano M., Alfieri O. (2020). Severe aortic stenosis in the young, with or without bicuspid valve: Is transcatheter aortic valve implantation the first choice?. Eur. Heart J. Suppl..

[11] Rudolph T., Appleby C., Delgado V., Eltchaninoff H., Gebhard C., Hengstenberg C., Wojakowski W., Petersen N., Kurucova J., Bramlage P. (2023). Patterns of Aortic Valve Replacement in Europe: Adoption by Age. Cardiology.

[12] Young M.N., Kearing S., Malenka D., Goodney P.P., Skinner J., Iribarne A. (2021). Geographic and Demographic Variability in Transcatheter Aortic Valve Replacement Dispersion in the United States. J. Am. Heart Assoc..

[13] Généreux P., Piazza N., Alu M.C., Nazif T., Hahn R.T., Pibarot P., Bax J.J., A Leipsic J., Blanke P., VARC-3 Writing Committee (2021). Valve Academic Research Consortium 3: Updated endpoint definitions for aortic valve clinical research. Eur. Heart J..

[14] Havers-Borgersen E., Butt J.H., Vinding N.E., Torp-Pedersen C., Gislason G., Køber L., Fosbøl E.L. (2020). Time in therapeutic range and risk of thromboembolism and bleeding in patients with a mechanical heart valve prosthesis. J. Thorac. Cardiovasc. Surg..

[15] Zhu Z., Li Y., Meng X., Han J., Li Y., Liu K., Shen J., Qin Y., Zhang H. (2019). New warfarin anticoagulation management model after heart valve surgery: Rationale and design of a prospective, multicentre, randomised trial to compare an internet-based warfarin anticoagulation management model with the traditional warfarin management model. BMJ Open.

[16] Ludwig S., Klimek M., Bay B., Blankenberg S., Granada J.F., Hildick-Smith D., Hudson J., Jørgensen T.H., Leon M.B., Magnussen C. (2025). Transcatheter or Surgical Treatment of Patients with Aortic Stenosis at Low to Intermediate Risk: An Individual Participant Data Meta-Analysis. JAMA Cardiol..

[17] Mack M.J., Leon M.B., Thourani V.H., Pibarot P., Hahn R.T., Genereux P., Kodali S.K., Kapadia S.R., Cohen D.J., Pocock S.J. (2023). Transcatheter Aortic-Valve Replacement in Low-Risk Patients at Five Years. N. Engl. J. Med..

[18] Thyregod H.G.H., Ihlemann N., Jørgensen T.H., Nissen H., Kjeldsen B.J., Petursson P., Chang Y., Franzen O.W., Engstrøm T., Clemmensen P. (2019). Five-Year Clinical and Echocardiographic Outcomes from the NOTION Randomized Clinical Trial in Patients at Lower Surgical Risk. Circulation.

[19] Forrest J.K., Yakubov S.J., Deeb G.M., Gada H., Mumtaz M.A., Ramlawi B., Bajwa T., Crouch J., Merhi W., Sang S.L.W. (2025). 5-Year Outcomes After Transcatheter or Surgical Aortic Valve Replacement in Low-Risk Patients with Aortic Stenosis. J. Am. Coll. Cardiol..

[20] Jørgensen T.H., Thyregod H.G.H., Savontaus M., Willemen Y., Bleie Ø., Tang M., Niemela M., Angerås O., Gudmundsdóttir I.J., Sartipy U. (2024). Transcatheter aortic valve implantation in low-risk tricuspid or bicuspid aortic stenosis: The NOTION-2 trial. Eur. Heart J..

[21] Jørgensen T.H., Savontaus M., Willemen Y., Bleie Ø., Tang M., Angerås O., Niemela M., Gudmundsdóttir I.J., Khokhar A., Sartipy U. (2025). Three-Year Follow-Up of the NOTION-2 Trial: TAVR Versus SAVR to Treat Younger Low-Risk Patients With Tricuspid or Bicuspid Aortic Stenosis. Circulation.

[22] Alabbadi S., Bowdish M.E., Sallam A., Tam D.Y., Hasan I., Kumaresan A., Alzahrani A.H., Iribarne A., Egorova N., Chikwe J. (2025). Transcatheter versus surgical aortic valve replacement in patients younger than 65 years in the United States. J. Thorac. Cardiovasc. Surg..

[23] Alabbadi S., Malas J., Chen Q., Cheng W., Tam D.Y., Cohen R.G., Bowdish M.E., Egorova N., Chikwe J. (2025). Guidelines vs Practice: Surgical Versus Transcatheter Aortic Valve Replacement in Adults ≤60 Years. Ann. Thorac. Surg..

[24] Gupta T., DeVries J.T., Gilani F., Hassan A., Ross C.S., Dauerman H.L. (2024). Temporal Trends in Transcatheter Aortic Valve Replacement for Isolated Severe Aortic Stenosis. J. Soc. Cardiovasc. Angiogr. Interv..

[25] Sohn S.H., Kim K.H., Kang Y., Choi J.W., Lee S.H., Shinn S.H., Yoo J.S. (2025). National Midterm Outcomes of Transcatheter Aortic Valve Implantation vs Surgical Aortic Valve Replacement. Ann. Thorac. Surg. Short. Rep..

[26] Brízido C., Madeira M., Brito J., Madeira S., Teles R.C., Raposo L., Gabriel H.M., Nolasco T., Gonçalves P.D.A., Sousa-Uva M. (2021). Surgical versus transcatheter aortic valve replacement in low-risk patients: A long-term propensity score-matched analysis. Catheter. Cardiovasc. Interv..

[27] Hazique M., Jafar Z., Lohana S., Reyaz I., Burhan M., Narayan R., Alraies M.C. (2025). The cusp overlap technique reduces pacemaker implantation in TAVR—A systematic review and meta-analysis. Am. J. Cardiol..

[28] Synetos A., Ktenopoulos N., Katsaros O., Vlasopoulou K., Drakopoulou M., Koliastasis L., Kachrimanidis I., Apostolos A., Tsalamandris S., Latsios G. (2025). Paravalvular Leak in Transcatheter Aortic Valve Implantation: A Review of Current Challenges and Future Directions. J. Cardiovasc. Dev. Dis..

