# Supplementary material for: Transcatheter Versus Mechanical and Bioprosthetic Surgical Aortic Valve Replacement in Retrospective Patient Cohorts with Aortic Stenosis <75 Years
Source: J Clin Med. 2026 Jul 16;15(14):5574. doi: 10.3390/jcm15145574 (PMC13412188; doi:10.3390/jcm15145574)
Supplement: Supplementary file 1 [file jcm-15-05574-s001.zip › jcm-4411178-supplementary.pdf]

Supplementary Figure S1

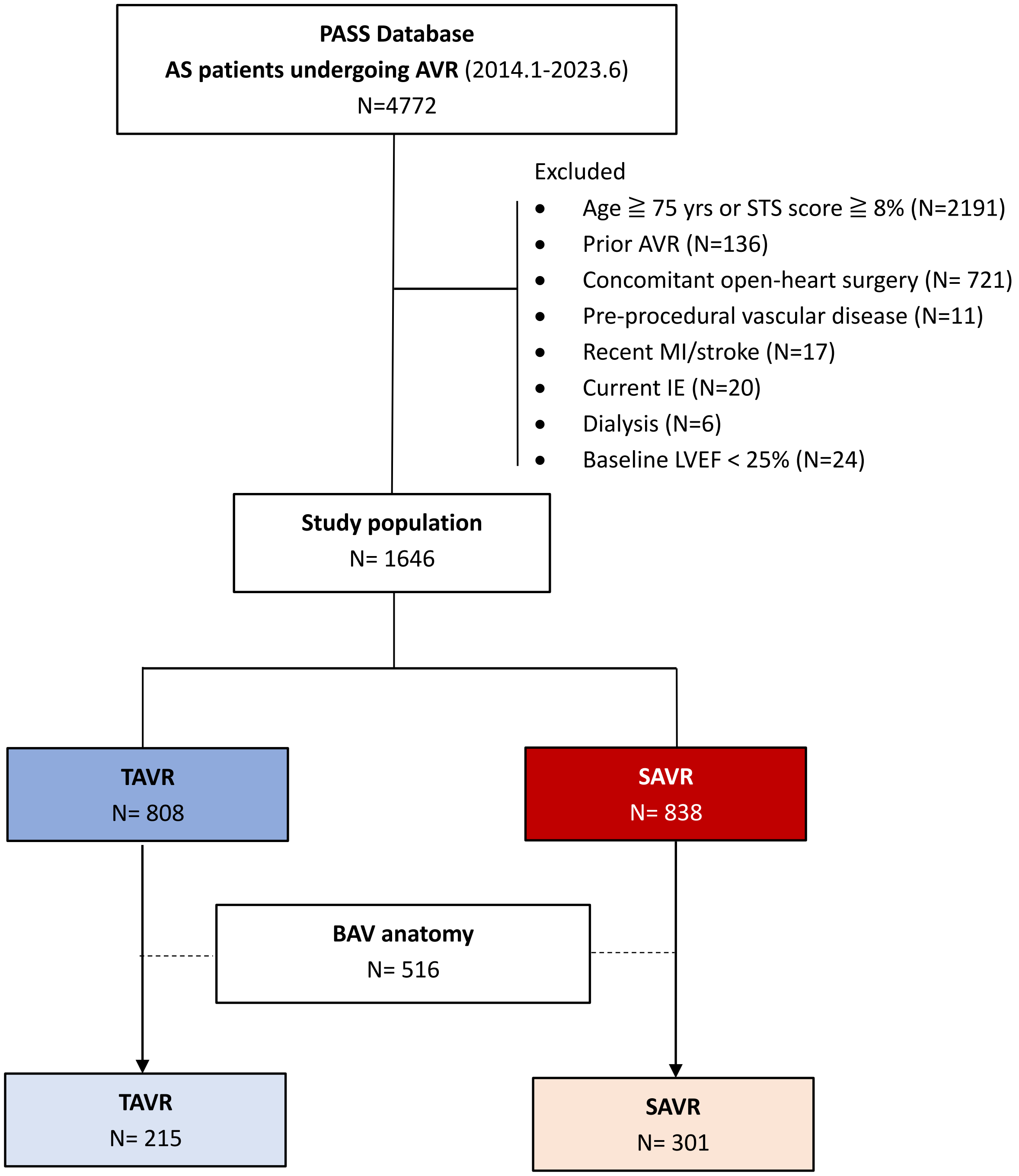

Flow chart of the included patient population. AS, aortic stenosis; AVR, aortic valve replacement; BAV, bicuspid aortic valve; IE, infective endocarditis; LVEF, left ventricular ejection fraction; MI, myocardial infarction; PASS, Percutaneous or surgical treatment of heart valves; SAVR, surgical aortic valve replacement; STS, Society of Thoracic Surgeons; TAVR, transcatheter aortic valve replacement.

Supplementary Figure S2

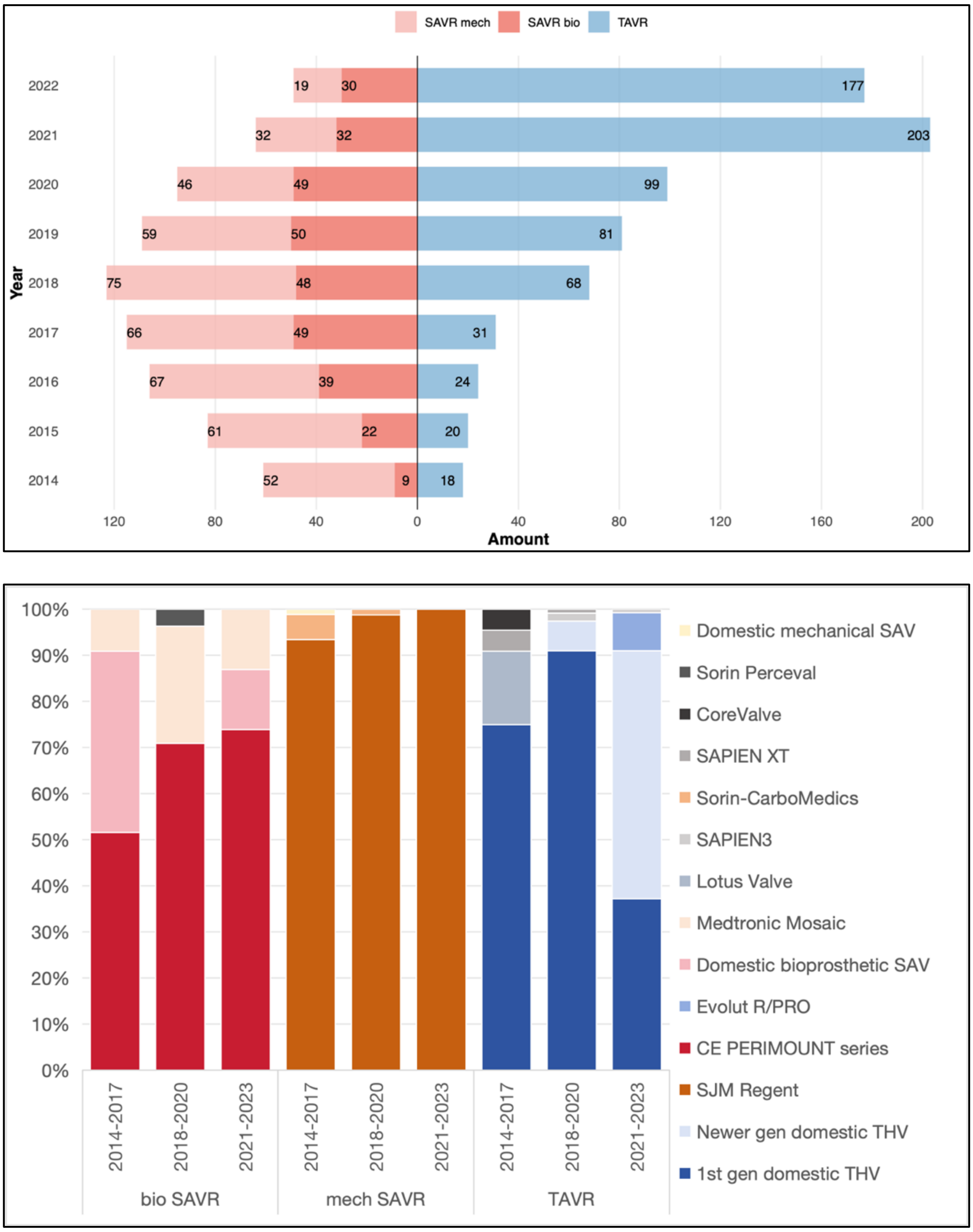

Clinical practice change from 2014 to 2022 in AS patients < 75 years. Over the study period, the incidence of TAVR use in AS patients < 75 years increased from 23% in 2014 to 78% in 2022. While the use of surgical prosthesis stayed similar over the study period, most TAVR patients received the newer generation of domestic device after 2021. SAV, surgical aortic valve; SAVR, surgical aortic valve replacement; TAVR, transcatheter aortic valve replacement; THV, transcatheter heart valve.

Supplementary Figure S3

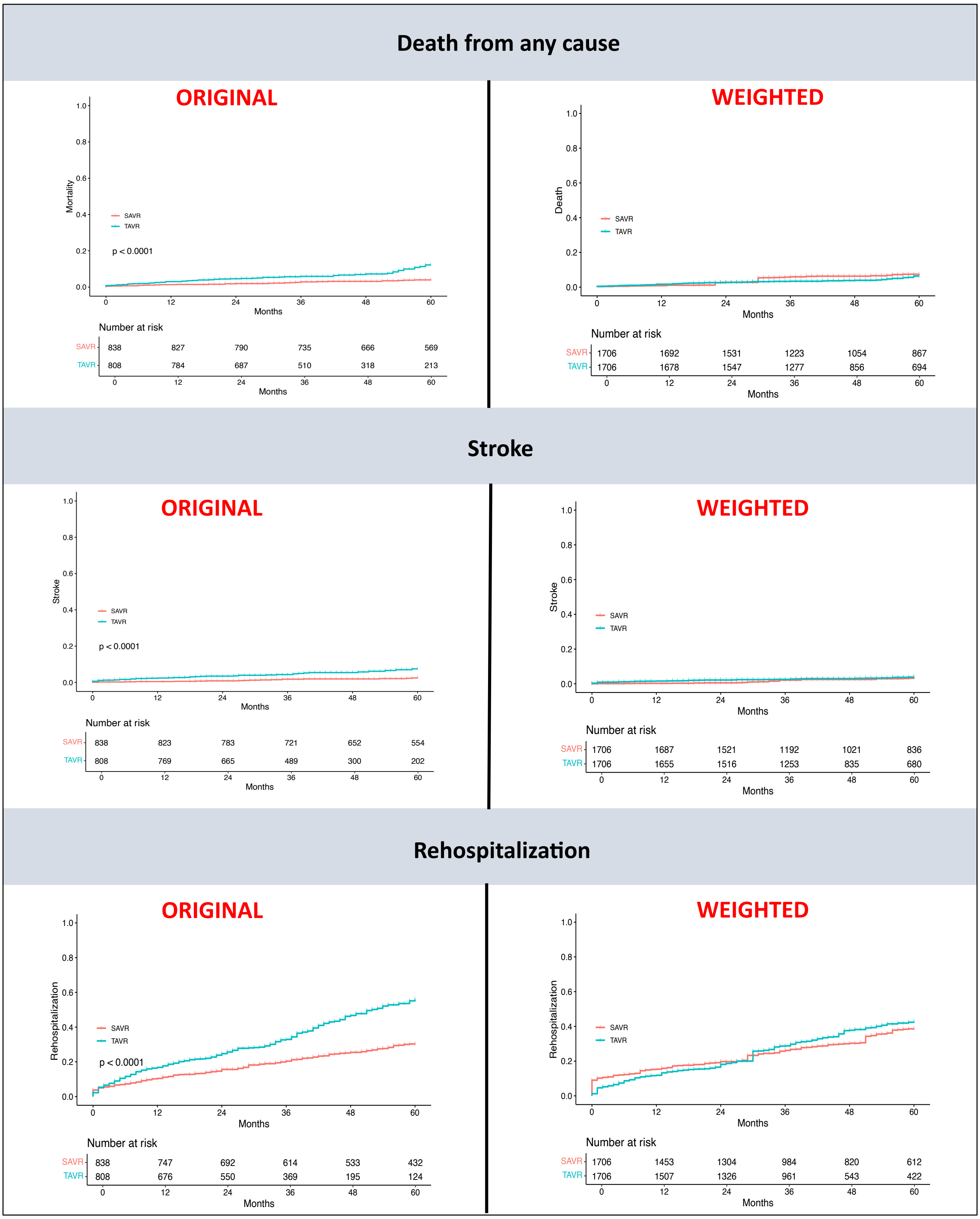

The original KM risk curves and the weighted risk curves for major clinical outcomes in the overall cohort. Before weighting, the risk of the outcomes was significantly higher in the TAVR group than the SAVR group in the overall cohort (Log-rank test, all p<0.01). After weighting, the weighted risk of the outcomes was similar between the two groups. SAVR, surgical aortic valve replacement; TAVR, transcatheter aortic valve replacement.

Supplementary Figure S4

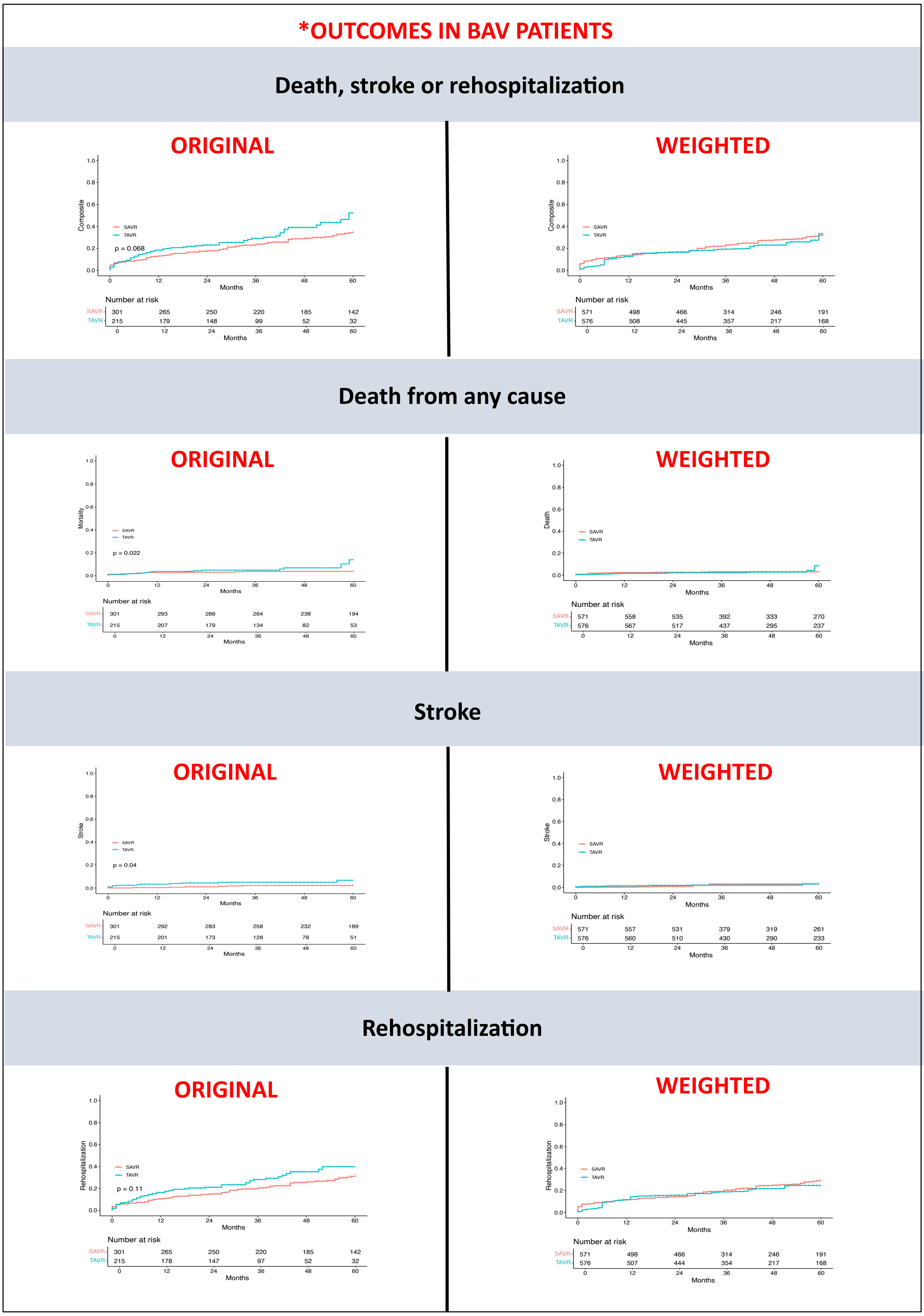

The original KM risk curves and the weighted risk curves for major clinical outcomes in patients with BAV anatomy. After weighting, the weighted risk of the outcomes was similar between the two groups in patients with BAV anatomy. BAV, bicuspid aortic valve; SAVR, surgical aortic valve replacement; TAVR, transcatheter aortic valve replacement.

Supplementary Figure S5

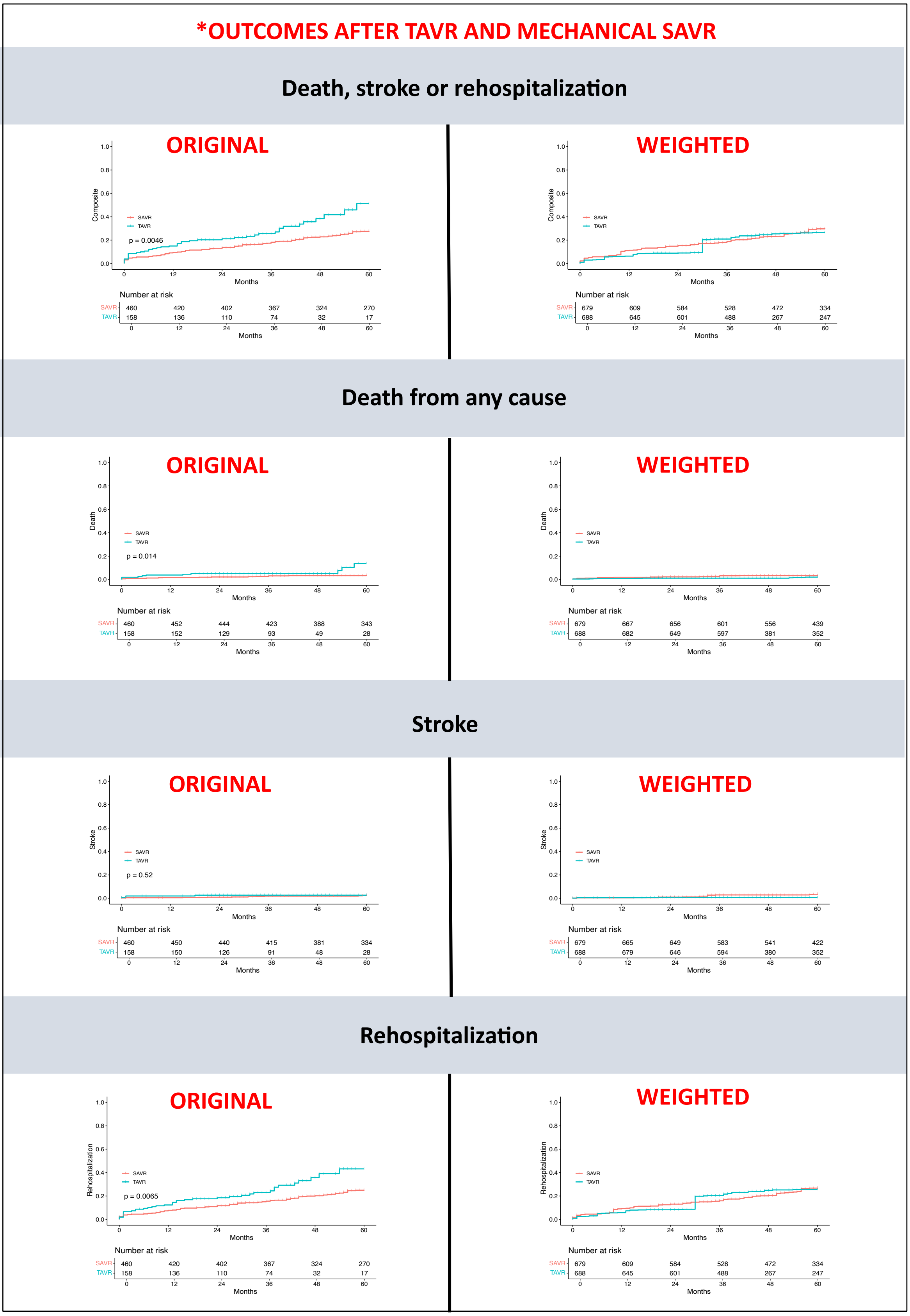

The original KM risk curves and the weighted risk curves for major clinical outcomes after TAVR and mechanical SAVR. After weighting, the weighted risk of the primary outcome was comparable between TAVR and mechanical SAVR groups, while the risk of stroke was higher in mechanical SAVR group. SAVR, surgical aortic valve replacement; TAVR, transcatheter aortic valve replacement.

Supplementary Figure S6

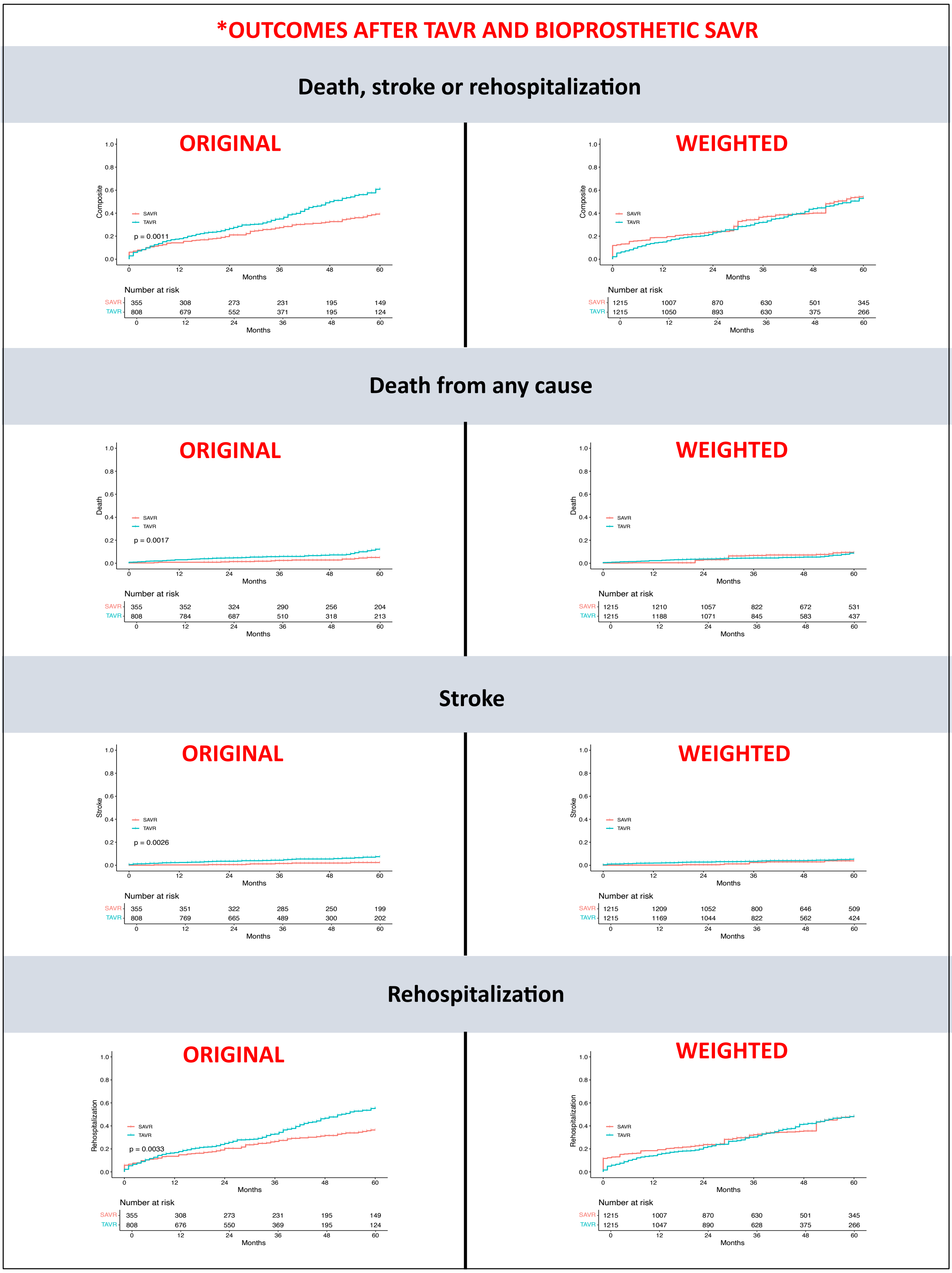

The original KM risk curves and the weighted risk curves for major clinical outcomes after TAVR and bioprosthetic SAVR. After weighting, the weighted risk of all outcomes was similar between the TAVR and bioprosthetic SAVR groups. SAVR, surgical aortic valve replacement; TAVR, transcatheter aortic valve replacement.

Supplementary Figure S7

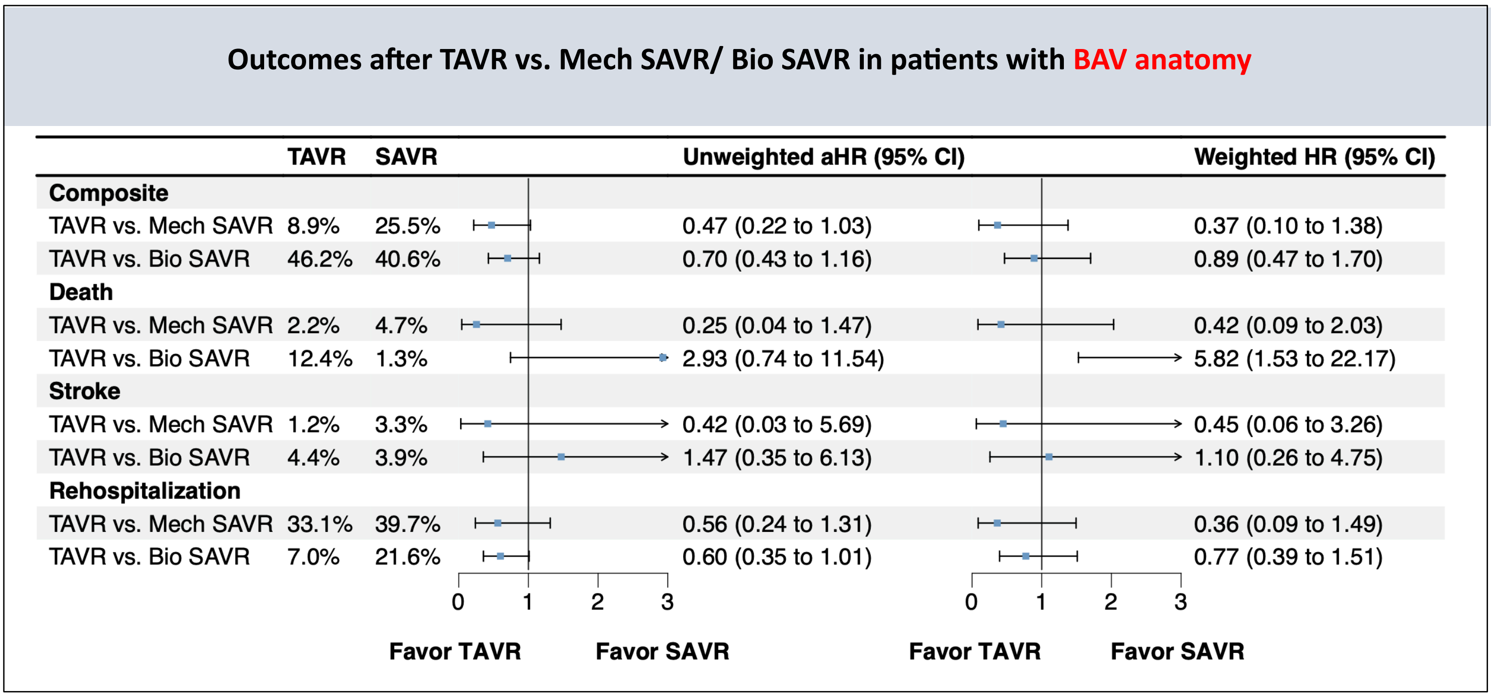

Outcomes after TAVR vs. Mech SAVR/ Bio SAVR in patients with BAV anatomy

	TAVR	SAVR	Unweighted aHR (95% CI)	Weighted HR (95% CI)
**Composite**				
TAVR vs. Mech SAVR	8.9%	25.5%	0.47 (0.22 to 1.03)	0.37 (0.10 to 1.38)
TAVR vs. Bio SAVR	46.2%	40.6%	0.70 (0.43 to 1.16)	0.89 (0.47 to 1.70)
**Death**				
TAVR vs. Mech SAVR	2.2%	4.7%	0.25 (0.04 to 1.47)	0.42 (0.09 to 2.03)
TAVR vs. Bio SAVR	12.4%	1.3%	2.93 (0.74 to 11.54)	5.82 (1.53 to 22.17)
**Stroke**				
TAVR vs. Mech SAVR	1.2%	3.3%	0.42 (0.03 to 5.69)	0.45 (0.06 to 3.26)
TAVR vs. Bio SAVR	4.4%	3.9%	1.47 (0.35 to 6.13)	1.10 (0.26 to 4.75)
**Rehospitalization**				
TAVR vs. Mech SAVR	33.1%	39.7%	0.56 (0.24 to 1.31)	0.36 (0.09 to 1.49)
TAVR vs. Bio SAVR	7.0%	21.6%	0.60 (0.35 to 1.01)	0.77 (0.39 to 1.51)

Weighted major clinical outcomes after TAVR vs. Mechanical SAVR and vs. Bioprosthetic SAVR in patients with BAV anatomy. In patients with BAV anatomy, the weighted risk for all outcomes was comparable between TAVR and mechanical SAVR group, while the weighted risk of all-cause death was significantly higher in TAVR group than bioprosthetic SAVR group (12.4% vs. 1.3%, weighted HR, 5.82; 95% CI, 1.53-22.17; p=0.010). BAV, bicuspid aortic valve; SAVR, surgical aortic valve replacement; TAVR, transcatheter aortic valve replacement.

Supplementary Figure S8

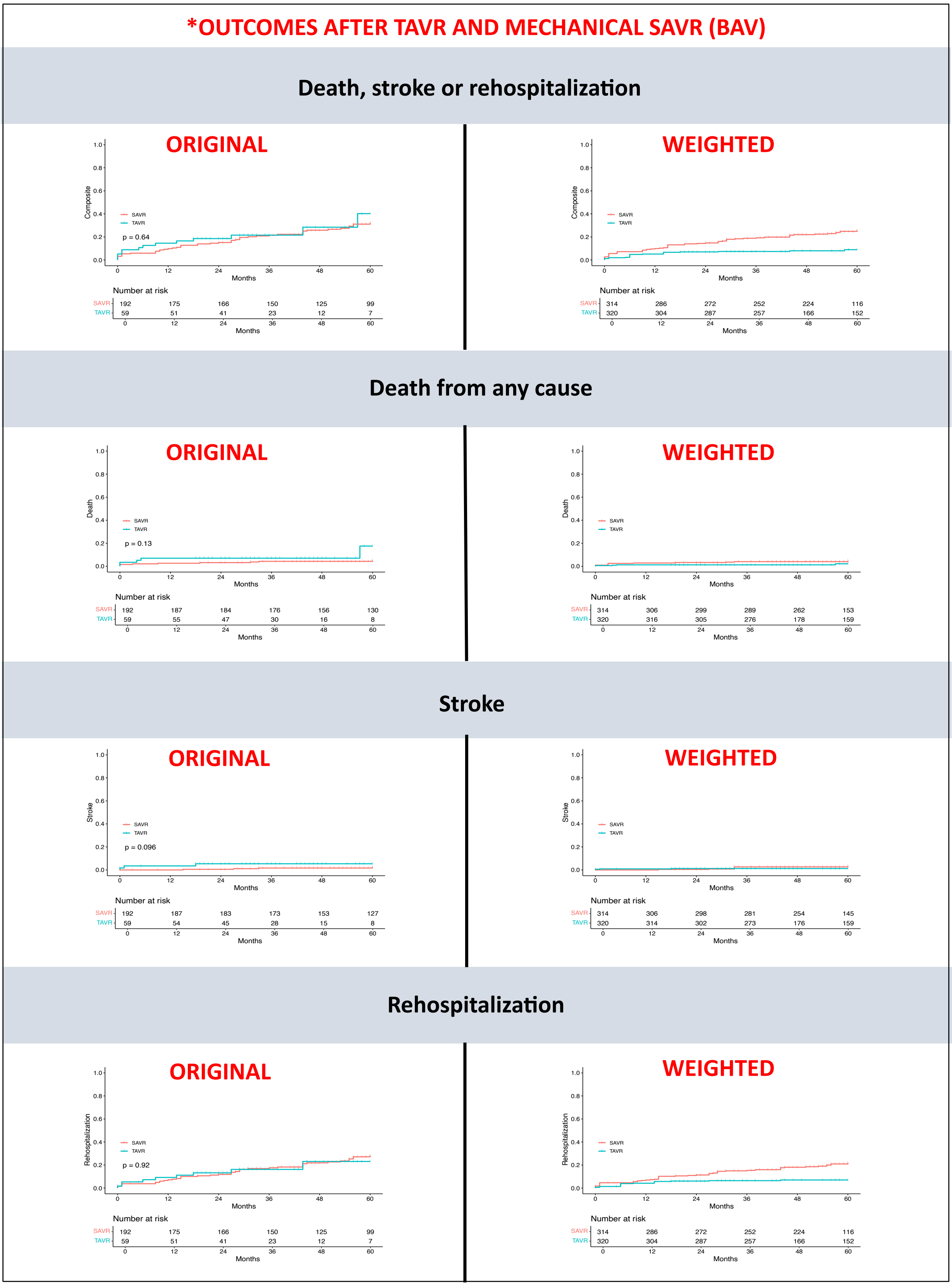

The original KM risk curves and the weighted risk curves for major clinical outcomes after TAVR and mechanical SAVR (BAV). After weighting, the risk of all outcomes was comparable between the TAVR group and the mechanical SAVR group in patients < 65 years with BAV anatomy. BAV, bicuspid aortic valve; SAVR, surgical aortic valve replacement; TAVR, transcatheter aortic valve replacement.

Supplementary Figure S9

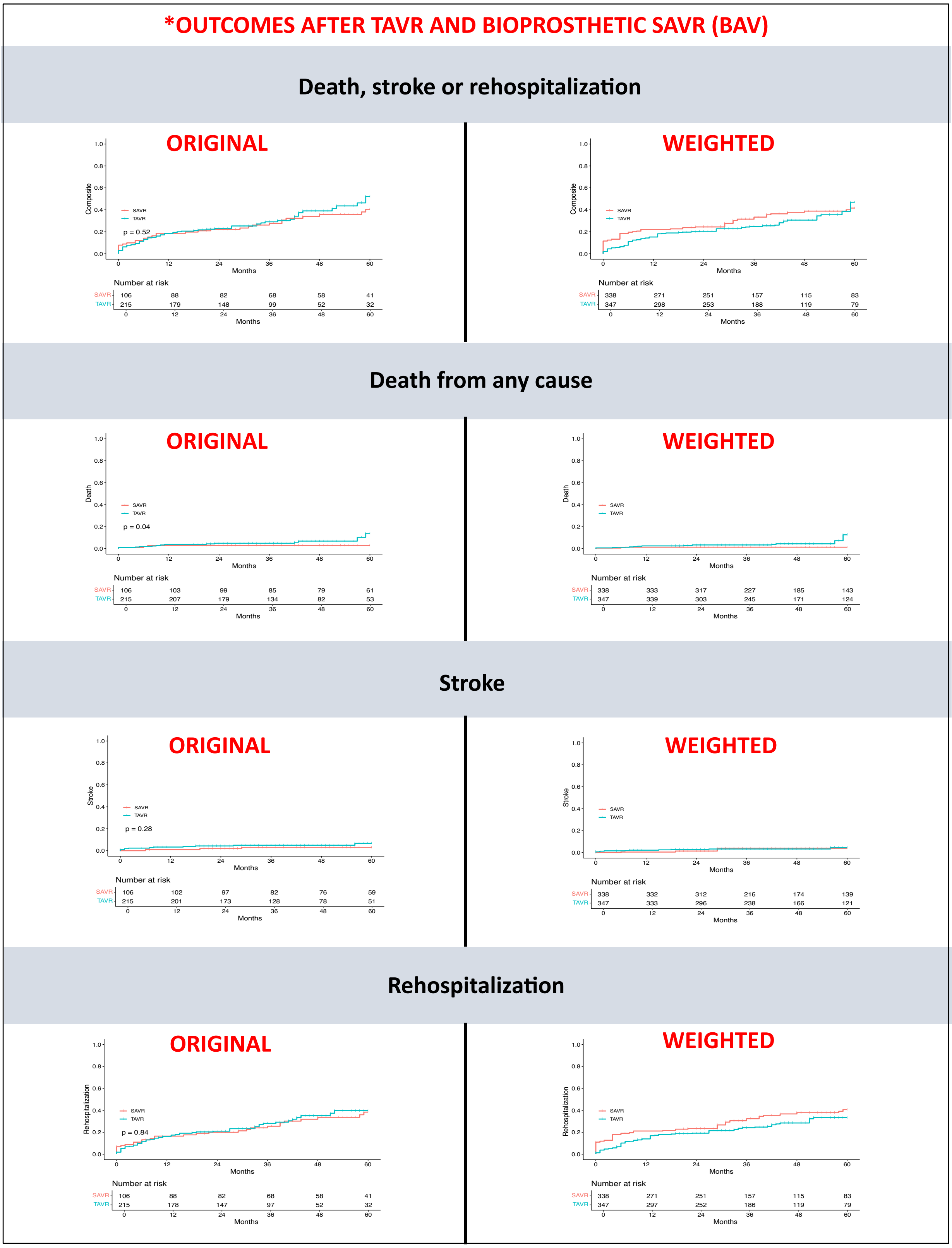

The original KM risk curves and the weighted risk curves for major clinical outcomes after TAVR and bioprosthetic SAVR (BAV). After weighting, the weighted risk of all-cause death was still higher in the TAVR group (p=0.010), while the risk of other outcomes was similar between the two groups. BAV, bicuspid aortic valve; SAVR, surgical aortic valve replacement; TAVR, transcatheter aortic valve replacement.

Supplementary Table S1. Baseline characteristics between the TAVR and the SAVR group in patients with BAV anatomy, before and after weighting.

	Unweighted			IPTW-Weighted		
	TAVR (n=215)	SAVR (n=301)	SMD	TAVR	SAVR	SMD
Age, years	66.9 ± 6.4	54.0 ± 11.0	1.45	60.0 ± 12.3	60.1 ± 10.7	<0.01
Male	111 (51.6)	151 (50.2)	0.03	54.8	54.6	<0.01
BMI, kg/m^2	23.7 ± 3.4	23.7 ± 3.1	<0.01	23.8 ± 2.9	23.8 ± 3.0	0.02
STS score, %	2.9 ± 1.4	2.0 ± 0.9	0.71	2.3 ± 1.1	2.3 ± 1.0	0.001
Hypertension	71 (33.0)	55 (18.3)	0.34	25.9	25.3	0.01
Diabetes mellitus	37 (17.2)	17 (5.6)	0.37	7.7	7.3	0.02
COPD	31 (14.4)	1 (0.3)	0.56	5.4	4.9	0.02
Coronary artery disease	35 (16.3)	23 (7.6)	0.27	11.1	10.5	0.02
Peri-artery disease	7 (3.3)	0 (0.0)	0.26	1.2	0.0	0.16
Prior stroke	7 (3.3)	3 (1.0)	0.16	1.5	1.1	0.03
Atrial fibrillation	15 (7.0)	4 (1.3)	0.29	2.9	2.6	0.02
Chronic kidney disease	15 (7.0)	1 (0.3)	0.36	2.8	2.4	0.02
Cancer	6 (2.8)	2 (0.7)	0.16	1.4	1.0	0.03
LVEF, %	58.3 ± 13.6	65.1 ± 9.4	0.59	58.6 ± 14.8	58.8 ± 12.9	0.01
Concomitant CABG/PCI	4 (1.9)	11 (3.7)	0.11	5.0	4.7	0.01

CABG, coronary artery bypass grafting; COPD, chronic obstructive pulmonary disease; BAV, bicuspid aortic valve; BMI, body mass index; IPTW, inverse probability of treatment weighting; LVEF, left ventricular ejection fraction; PCI, percutaneous coronary intervention; SAVR, surgical aortic valve replacement; SMD, standardized mean difference; STS, Society of Thoracic Surgeons; TAVR, transcatheter aortic valve replacement.

Supplementary Table S2. Key secondary endpoints at 5 years between TAVR and SAVR group in patients with BAV anatomy.

	Cumulative risk over 5 years, %		Weighted risk over 5 years, %		Weighted HR (95% CI)	P value
	TAVR N = 215	SAVR N = 301	TAVR	SAVR		
Heart failure rehospitalizations	19.0	7.4	10.5	6.5	1.64 (0.53-5.11)	0.391
Procedure related rehospitalizations	12.6	5.8	5.3	5.9	0.97 (0.39-2.41)	0.949
Valve related rehospitalizations	2.4	5.9	5.2	6.5	1.10 (0.20-5.95)	0.916
Other CV rehospitalizations	5.5	10.7	5.0	9.2	0.60 (0.18-2.00)	0.404
Bleeding	2.4	5.3	5.2	4.9	1.70 (0.32-9.18)	0.535
New permanent pacemaker implant	15.8	3.0	8.4	2.1	5.19 (2.04-13.16)	<0.001
Moderate/severe paravalvular leak	8.8	0	26.0	0		<0.001

BAV, bicuspid aortic valve; CI, confidence interval; CV, cardiovascular; HR, hazard ratio; SAVR, surgical aortic valve replacement; TAVR, transcatheter aortic valve replacement.

Supplementary Table S3. Baseline characteristics between the TAVR and the mechanical SAVR group (in patients < 65yrs), before and after weighting.

	Unweighted			IPTW-Weighted		
	TAVR (n=158)	SAVR (n=460)	SMD	TAVR	SAVR	SMD
Age, years	59.9 ± 5.2	50.9 ± 8.4	1.30	53.6 ± 12.5	53.8 ± 8.4	0.02
Male	93 (58.9)	223 (48.5)	0.21	55.6	54.8	<0.01
BMI, kg/m^2	23.3 ± 3.6	23.9 ± 3.2	0.20	24.0 ± 3.2	24.1 ± 3.0	0.04
STS score, %	2.6 ± 1.5	2.0 ± 0.9	0.53	2.0 ± 0.9	2.0 ± 0.9	0.02
Hypertension	46 (29.1)	67 (14.6)	0.36	20.3	19.5	0.02
Diabetes mellitus	29 (18.4)	20 (4.3)	0.45	5.6	4.7	0.04
COPD	16 (10.1)	1 (0.2)	0.46	3.1	2.5	0.04
Coronary artery disease	19 (12.0)	18 (3.9)	0.30	7.4	6.7	0.03
Peri-artery disease	5 (3.2)	0 (0.0)	0.26	0.7	0.0	0.12
Prior stroke	4 (2.5)	4 (0.9)	0.13	1.8	1.3	0.04
Atrial fibrillation	14 (8.9)	10 (2.2)	0.30	2.7	2.1	0.04
Chronic kidney disease	11 (7.0)	0 (0.0)	0.39	1.6	0.0	0.18
Cancer	6 (3.8)	1 (0.2)	0.26	0.9	0.3	0.07
LVEF, %	53.5 ± 16.2	64.1 ± 9.6	0.80	58.7 ± 17.3	59.3 ± 12.2	0.04
Concomitant CABG/PCI	11 (7.0)	8 (1.7)	1.30	2.7	2.2	0.03

CABG, coronary artery bypass grafting; COPD, chronic obstructive pulmonary disease; BMI, body mass index; IPTW, inverse probability of treatment weighting; LVEF, left ventricular ejection fraction; PCI, percutaneous coronary intervention; SAVR, surgical aortic valve replacement; SMD, standardized mean difference; STS, Society of Thoracic Surgeons; TAVR, transcatheter aortic valve replacement.

Supplementary Table S4. Baseline characteristics between the TAVR and the bioprosthetic SAVR group, before and after weighting.

	Unweighted			IPTW-Weighted		
	TAVR (n=808)	SAVR (n=355)	SMD	TAVR	SAVR	SMD
Age, years	67.9 ± 5.2	63.0 ± 5.7	0.88	65.8 ± 7.3	65.8 ± 5.2	<0.001
Male	440 (54.5)	187 (52.7)	0.04	53.6	53.6	<0.001
BMI, kg/m^2	23.3 ± 3.6	23.8 ± 3.2	0.16	23.8 ± 3.6	23.8 ± 3.4	<0.001
STS score, %	3.1 ± 1.5	2.3 ± 1.0	0.59	2.7 ± 1.4	2.8 ± 1.3	<0.001
Hypertension	288 (35.6)	94 (26.5)	0.20	33.3	33.3	<0.001
Diabetes mellitus	139 (17.2)	26 (7.3)	0.31	14.2	14.2	<0.001
COPD	140 (17.3)	7 (2.0)	0.54	11.8	11.8	<0.001
Coronary artery disease	168 (20.8)	46 (13.0)	0.21	19.0	19.0	<0.001
Peri-artery disease	22 (2.7)	1 (0.3)	0.20	2.5	2.5	<0.001
Prior stroke	30 (3.7)	3 (0.8)	0.19	2.6	2.6	<0.001
Atrial fibrillation	94 (11.6)	10 (2.8)	0.35	8.3	8.3	<0.001
Chronic kidney disease	55 (6.8)	5 (1.4)	0.28	4.7	4.7	<0.001
Cancer	20 (2.5)	3 (0.8)	0.13	1.7	1.7	<0.001
LVEF, %	57.0 ± 14.0	62.6 ± 10.4	0.45	58.5 ± 14.2	58.5 ± 12.0	<0.001
Concomitant CABG/PCI	11 (1.4)	20 (5.6)	0.23	6.7	6.7	<0.001

CABG, coronary artery bypass grafting; COPD, chronic obstructive pulmonary disease; BMI, body mass index; IPTW, inverse probability of treatment weighting; LVEF, left ventricular ejection fraction; PCI, percutaneous coronary intervention; SAVR, surgical aortic valve replacement; SMD, standardized mean difference; STS, Society of Thoracic Surgeons; TAVR, transcatheter aortic valve replacement.

Supplementary Table S5. Key secondary endpoints at 5 years between TAVR and mechanical or bioprosthetic SAVR group.

	Cumulative risk over 5 years, %		Weighted risk over 5 years, %		Weighted HR (95% CI)	P value
	TAVR	SAVR	TAVR	SAVR		
Heart failure rehospitalizations						
TAVR vs. mech SAVR	16.7	6.3	6.0	9.3	0.70 (0.20-2.50)	0.586
TAVR vs. bio SAVR	23.8	8.9	17.6	10.7	1.35 (0.69-2.66)	0.380
Procedure related rehospitalizations						
TAVR vs. mech SAVR	10.9	4.9	11.9	7.2	1.69 (0.28-10.31)	0.572
TAVR vs. bio SAVR	14.1	8.11	12.5	16.7	0.87 (0.42-1.79)	0.702
Valve related rehospitalizations						
TAVR vs. mech SAVR	2.6	5.3	2.4	6.4	0.50 (0.08-2.93)	0.440
TAVR vs. bio SAVR	8.2	5.0	6.4	6.2	0.95 (0.41-2.17)	0.898
Other CV rehospitalizations						
TAVR vs. mech SAVR	12.6	8.8	3.8	8.2	0.49 (0.17-1.41)	0.184
TAVR vs. bio SAVR	8.2	13.1	8.9	10.9	0.76 (0.44-1.31)	0.322
Bleeding						
TAVR vs. mech SAVR	2.6	5.1	2.4	6.3	0.51 (0.09-3.02)	0.460
TAVR vs. bio SAVR	7.5	2.5	5.8	3.2	1.87 (0.63-5.55)	0.261
New permanent pacemaker implant						
TAVR vs. mech SAVR	11.0	1.6	27.2	1.2	6.94 (1.97-24.41)	0.002
TAVR vs. bio SAVR	18.2	2.8	21.7	3.9	27.7 (5.6-133.2)	<0.001
Moderate/severe paravalvular leak						
TAVR vs. mech SAVR	8.7	0.2	6.6	0.2	39.79 (3.43-461.06)	0.003
TAVR vs. bio SAVR	6.1	0	7.1	0		<0.001

CI, confidence interval; CV, cardiovascular; HR, hazard ratio; SAVR, surgical aortic valve replacement; TAVR, transcatheter aortic valve replacement.

Supplementary Table S6. Baseline characteristics between the TAVR and the mechanical SAVR group in patients with BAV anatomy (< 65yrs), before and after weighting.

	Unweighted			IPTW-Weighted		
	TAVR (n=59)	SAVR (n=192)	SMD	TAVR	SAVR	SMD
Age, years	59.1 ± 6.1	49.1 ± 9.9	1.21	53.4 ± 13.3	53.6 ± 10.0	0.01
Male	36 (61.0)	94 (49.0)	0.24	61.2	60.5	0.01
BMI, kg/m^2	23.7 ± 3.7	23.7 ± 3.1	<0.01	23.5 ± 2.4	23.6 ± 2.7	0.06
STS score, %	2.5 ± 1.5	1.9 ± 0.9	0.44	2.0 ± 0.8	2.0 ± 0.9	0.02
Hypertension	15 (25.4)	26 (13.5)	0.30	12.8	12.1	0.02
Diabetes mellitus	10 (16.9)	11 (5.7)	0.36	7.5	6.5	0.04
COPD	5 (8.5)	0 (0.0)	0.43	1.6	0.0	0.18
Coronary artery disease	9 (15.3)	9 (4.7)	0.36	7.4	6.4	0.04
Peri-artery disease	2 (3.4)	0 (0.0)	0.27	0.6	0.0	0.11
Prior stroke	1 (1.7)	2 (1.0)	0.06	2.6	2.0	0.04
Atrial fibrillation	3 (5.1)	2 (1.0)	0.24	1.4	0.6	0.08
Chronic kidney disease	4 (6.8)	0 (0.0)	0.38	1.3	0.0	0.16
Cancer	2 (3.4)	1 (0.5)	0.21	0.9	0.3	0.08
LVEF, %	56.0 ± 16.1	65.7 ± 9.6	0.74	57.4 ± 15.7	57.8 ± 14.2	0.03
Concomitant CABG/PCI	4 (6.8)	4 (2.1)	0.23	1.9	1.3	0.04

BAV, bicuspid aortic valve; BMI, body mass index; CABG, coronary artery bypass grafting; COPD, chronic obstructive pulmonary disease; IPTW, inverse probability of treatment weighting; LVEF, left ventricular ejection fraction; PCI, percutaneous coronary intervention; SAVR, surgical aortic valve replacement; SMD, standardized mean difference; STS, Society of Thoracic Surgeons; TAVR, transcatheter aortic valve replacement.

Supplementary Table S7. Baseline characteristics between the TAVR and the bioprosthetic SAVR group in patients with BAV anatomy, before and after weighting.

	Unweighted			IPTW-Weighted		
	TAVR (n=215)	SAVR (n=106)	SMD	TAVR	SAVR	SMD
Age, years	66.9 ± 6.4	62.6 ± 6.5	0.68	64.6 ± 8.3	64.9 ± 5.4	0.04
Male	111 (51.6)	55 (51.9)	0.005	52.4	52.1	0.007
BMI, kg/m^2	23.7 ± 3.4	23.8 ± 3.2	0.03	23.8 ± 3.3	24.0 ± 3.0	0.04
STS score, %	2.9 ± 1.4	2.3 ± 1.0	0.52	2.6 ± 1.2	2.6 ± 1.1	0.002
Hypertension	71 (33.0)	29 (27.4)	0.12	31.5	30.6	0.02
Diabetes mellitus	37 (17.2)	6 (5.7)	0.37	11.3	10.6	0.02
COPD	31 (14.4)	1 (0.9)	0.52	9.0	7.8	0.04
Coronary artery disease	35 (16.3)	14 (13.2)	0.09	13.6	12.4	0.03
Peri-artery disease	7 (3.3)	0 (0.0)	0.26	2.0	0.0	0.20
Prior stroke	7 (3.3)	0 (0.0)	0.26	2.0	0.0	0.20
Atrial fibrillation	15 (7.0)	2 (1.9)	0.25	4.6	4.1	0.03
Chronic kidney disease	15 (7.0)	1 (0.9)	0.31	4.4	3.7	0.04
Cancer	6 (2.8)	1 (0.9)	0.14	2.1	1.5	0.05
LVEF, %	58.3 ± 13.6	64.1 ± 9.0	0.51	58.4 ± 14.0	58.7 ± 12.0	0.02
Concomitant CABG/PCI	4 (1.9)	7 (6.6)	0.24	6.3	6.0	0.02

BAV, bicuspid aortic valve; BMI, body mass index; CABG, coronary artery bypass grafting; COPD, chronic obstructive pulmonary disease; IPTW, inverse probability of treatment weighting; LVEF, left ventricular ejection fraction; PCI, percutaneous coronary intervention; SAVR, surgical aortic valve replacement; SMD, standardized mean difference; STS, Society of Thoracic Surgeons; TAVR, transcatheter aortic valve replacement.

Supplementary Table S8. Key secondary endpoints at 5 years between TAVR and mechanical or bioprosthetic SAVR group in patients with BAV anatomy.

	Cumulative risk over 5 years, %		Weighted risk over 5 years, %		Weighted HR (95% CI)	P value
	TAVR (BAV)	SAVR (BAV)	TAVR (BAV)	SAVR (BAV)		
Heart failure rehospitalizations						
TAVR vs. mech SAVR	8.8	5.8	3.3	4.1	0.88 (0.13-6.09)	0.897
TAVR vs. bio SAVR	19.0	10.4	13.1	12.2	0.88 (0.30-2.60)	0.822
Procedure related rehospitalizations						
TAVR vs. mech SAVR	13.1	4.2	1.9	4.4	0.44 (0.07-2.53)	0.354
TAVR vs. bio SAVR	12.6	9.1	7.9	7.8	1.07 (0.40-2.86)	0.887
Valve related rehospitalizations						
TAVR vs. mech SAVR	3.7	6.1	2.8	7.1	0.51 (0.06-4.23)	0.532
TAVR vs. bio SAVR	2.4	5.9	4.2	5.4	0.98 (0.20-4.94)	0.982
Other CV rehospitalizations						
TAVR vs. mech SAVR	8.2	9.9	2.6	7.1	0.39 (0.08-2.07)	0.271
TAVR vs. bio SAVR	5.5	11.3	6.5	10.9	0.66 (0.18-2.37)	0.522
Bleeding						
TAVR vs. mech SAVR	3.7	6.1	2.8	7.1	0.51 (0.06-4.23)	0.532
TAVR vs. bio SAVR	2.4	4.2	4.2	3.0	2.65 (0.46-15.19)	0.278
New permanent pacemaker implant						
TAVR vs. mech SAVR	5.1	2.3	1.0	1.5	0.73 (0.12-4.36)	0.733
TAVR vs. bio SAVR	15.8	4.5	12.9	2.6	6.51 (2.14-19.86)	<0.001
Moderate/severe paravalvular leak						
TAVR vs. mech SAVR	10.6	0	40.3	0		<0.001
TAVR vs. bio SAVR	8.8	0	17.8	0		<0.001

BAV, bicuspid aortic valve; CI, confidence interval; CV, cardiovascular; HR, hazard ratio; SAVR, surgical aortic valve replacement; TAVR, transcatheter aortic valve replacement.
